# In Silico Assessment of SYNGAP1 Missense Variant Pathogenicity

**DOI:** 10.1155/humu/8234670

**Published:** 2026-11-08

**Authors:** Aliaa E. Ali, Henna-Riikka M. Otranen, Jukka V. Lehtonen, Michael J. Courtney, Olli T. Pentikäinen, Pekka A. Postila

**Affiliations:** ^1^ Institute of Biomedicine Integrative Physiology and Pharmacology Faculty of Medicine University of Turku Turku Finland utu.fi; ^2^ InFLAMES Research Flagship University of Turku Turku Finland utu.fi; ^3^ Structural Bioinformatics Laboratory Department of Natural and Health Sciences Åbo Akademi University Turku Finland abo.fi; ^4^ InFLAMES Research Flagship Åbo Akademi University Turku Finland abo.fi; ^5^ Neuronal Signalling Laboratory and Turku Screening Unit Turku Bioscience Centre University of Turku and Åbo Akademi University Turku Finland; ^6^ Aurlide Ltd. Turku Finland

**Keywords:** database, missense mutation, missense variant, pathogenicity prediction, SynGAP, SynGAP missense (SGM) server, *SYNGAP1*, *SYNGAP1*-related disorders (SRD)

## Abstract

SynGAP, a critical regulator at the postsynaptic density, is associated with Alzheimer′s disease, tumorigenesis, and most notably, *SYNGAP1*‐related disorders (SRD), which manifests as autism, epilepsy, and cognitive impairment. SRD is likely primarily caused by SynGAP missense (SGM) variants, whose pathogenicity is difficult to confirm experimentally. To address this issue, a wide range of structure‐ and/or sequence‐based as well as machine learning– and deep learning–based in silico predictors were benchmarked against clinically annotated SRD data. Folding stability calculations (e.g., Foldetta) and deep learning methods ESM1b and AlphaMissense showed the highest prediction accuracy under default settings. Applying optimized nondefault thresholds, especially with AlphaMissense, could further improve predictor performance. The predictions exhibited a strong structural pattern: Pathogenic variants were highly enriched within defined structural domains, particularly the GAP and PH domains, whereas variants located in nondomain and nonmotif regions were predominantly predicted to be benign. The verified phosphosites and predicted phase separation impacting variants occupy matching sections, including the protein′s N‐terminal region and the vicinity of putative SH3‐binding motif in the disordered regions. Thus, these regions collectively could act as phase separation‐promoting segments, complementing the protein′s trimerization‐coupled coiled‐coil domain. Finally, the SynGAP Missense (SGM) server was presented to support SynGAP variant assessment. The SGM server integrates a *Data Table* with top‐performing predictors, a *3D Viewer* for structure‐level assessment and AI‐generated lay summaries for all variants arising from single‐base substitutions, including those not yet observed in public databases.

## 1. Introduction

SynGAP, the synaptic GTPase‐activating protein, is highly abundant at the postsynaptic density (PSD) of forebrain neurons and is crucial for cognitive development and learning in early childhood (Figure 1) [4]. The *SYNGAP1* gene, also known as *RASA5* (Ras GTPase‐activating protein 5), belongs to the GAP1 subfamily [1] and encodes a large multidomain protein containing PH (pleckstrin homology), C2, GAP, and CC (coiled‐coil) domains (Figure 1a,b) [1, 4, 5]. Increasing variability, SynGAP also undergoes extensive alternative splicing, producing isoforms with alternate N‐ and C‐termini, internal exon skipping, and alternative start sites [4, 6, 7].

**Figure 1:**
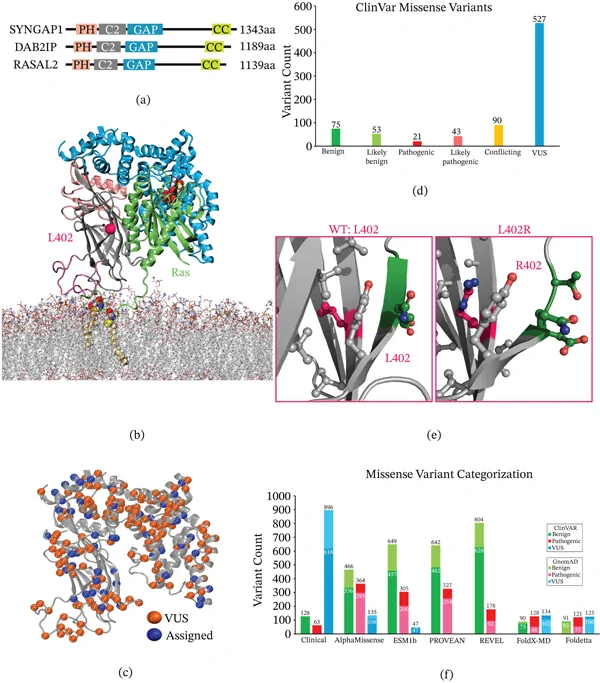
SynGAP domains, N‐terminal PH‐C2‐GAP model structure, and missense variants. (a) SynGAP subfamily proteins, including *SYNGAP1* or *RASA5*, *DAB2IP*, and *RASAL2* [1] contain C2 (protein kinase C2 homology; Res. 255–410, gray), PH (Plekstrin homology) (Res. 198–254, pink) and GAP (GTPase activating protein) (Res. 410–730, indigo) domains. The C‐terminal CC (coiled‐coil) (Res. 1170–1270) domain of SynGAP is also shown, but the C‐terminal T/SXV PDZ domain as well as other *DAB2IP* and *RASAL2* domains are omitted for clarity. Residue numbering is from *SYNGAP1.* (b) Based on the prior bioinformatics probe and modelling work [2], the protein (cartoon model) is shown in complex with both the inner postsynaptic membrane (stick models) and RasGTPase (green cartoon). (c) The already clinically assigned missense variants and VUS (variants of unclear significance) are shown as blue and orange spheres, respectively, on top of the N‐terminal SynGAP model. (d) Only a few SynGAP missense variants have been assigned benign or pathogenic status to date, and thus, a clear majority of variants are listed as conflicting or VUS in the ClinVar [3]. (e) The L402R is an example of missense variant that causes a substantial structural change based on the in silico mutagenesis analysis (pathogenic status in ClinVar) [3], although, in general, the protein fold was found to be resistant to large‐scale unfolding in the classical molecular dynamics simulations [2]. (f) By combining the ClinVar sets (benign = benign + likely benign; pathogenic = pathogenic + likely pathogenic; unclear = conflicting + VUS+ non‐ClinVar/gnomAD entries), enough noncontradictory or “established” data can be applied to the accuracy estimation. Indeed, the prediction methods typically categorize the variants into benign and pathogenic groups, whereas the uncertain (ambiguous, in‐between) category can also be included by default (e.g., ESM1b).

SynGAP is estimated to be largely intrinsically disordered outside the N‐terminal PH‐C2‐GAP region and the C‐terminal CC domain, which mediates trimerization (Figure S1 in Supporting Information, SI) [8]. Only short linear motifs within the disordered regions, including the NPx [F/Y] motif and the *α*1‐specific PDZ‐binding motif, that mediate interactions with scaffold proteins such as AIDA‐1 and PSD95 [9, 10] have been solved via X‐ray crystallography. These intrinsically disordered regions (IDRs), together with the CC domain, are likely to play key roles in liquid–liquid phase separation (LLPS) at the PSD, where scaffold and signaling proteins form dynamic, membraneless compartments [10, 11].

SynGAP is a dual Ras/Rap‐GTPase that negatively regulates the catalytic activity of both Ras‐ and Rap‐GTPases (Figure 1b) [12]. However, SynGAP provides a break to long‐term potentiation by limiting the trafficking of AMPA receptors (AMPAR) to the PSD, possibly through the Ras‐MAPK signaling pathway [13–15]. In contrast, SynGAP is released from the PSD after CaMKII phosphorylation by NMDA receptor activity [13, 16, 17]. Recently, SynGAP has also been suggested to modulate synaptic plasticity, independent of its GAP activity, by limiting the amount of PDZ domain binding slots available at the PSD for recruiting the AMPAR‐TARP (transmembrane AMPAR regulatory proteins) complexes [4, 17–20].

The importance of SynGAP in brain development is highlighted by the severe neurodevelopmental disorders associated with its dysfunction. De novo *SYNGAP1* variants disrupt synaptic plasticity and cause SynGAP‐related nonsyndromic intellectual disability or *SYNGAP1*‐related disorders (SRD) [21–23]. SRD manifests in early childhood and includes a broad spectrum of neurodevelopmental phenotypes, including epilepsy, autism spectrum disorder, and intellectual disability [24–28]. Although rare, with an estimated incidence of ~6 per 100,000 births [29, 30], *SYNGAP1* variants account for an estimated 1% of sporadic intellectual disability cases [23, 29] corresponding to a global patient population of roughly half a million individuals.

Diagnosis and variant interpretation remain challenging because SRD lacks a specific clinical profile [24, 31]. Moreover, pathogenicity is not limited to truncating variants that completely abolish protein production [32], but most cases are predicted to be caused by missense variants (Figure 1c,d; for example, L402R in Figure 1b,e) [29]. Missense variants are difficult to interpret from sequencing alone, especially in the absence of previous clinical reports, because their effects depend strongly on structural and functional context (Figure 1b,e) [2, 33, 34]. Currently, ~65% of SynGAP missense entries in ClinVar are classified as variants of uncertain significance (VUS), whereas only 15.8% and 7.9% are designated as benign/likely benign or pathogenic/likely pathogenic, respectively (Figure 1d) [3].

Robust experimental assays for assessing the pathogenicity of SynGAP missense variants are missing [6, 15]. Even when available for other diseases, such assays can be labor‐intensive, time‐consuming, and costly [35–38]. Consequently, computational prediction has become an essential component of missense variant interpretation. The predictors have progressed from traditional sequence‐based approaches (e.g., SIFT [39]) to ensemble methods (e.g., REVEL [40]) and, more recently, to deep learning (DL) and protein language model‐based predictors such as ESM1b [41] and AlphaMissense [42], which are among the computational predictors calibrated for ACMG/AMP PP3/BP4 evidence assessment [43]. However, prediction accuracy is often protein‐dependent (calibration needed), and relevant tools and datasets are scattered across standalone software, web servers, and online databases with varying usability and accessibility [44, 45]. This fragmentation complicates benchmarking and routine analysis, while providing limited disease‐specific support for disorders such as SRD.

ClinVar submissions of *SYNGAP1* missense variants continue to increase, with most remaining classified as VUS (Figure 1d). To explore strategies for improving variant annotation, established and emerging in silico predictors were benchmarked and, where possible, calibrated using a limited but carefully curated set of clinically annotated variants. These analyses supported the development of the SynGAP Missense (SGM) server, an online platform that integrates in silico pathogenicity predictions for known and all possible single‐base‐substitution‐derived missense variants including variant 3D models (e.g., molecular simulation snapshots from a prior study [46]) and AI‐generated prediction summaries. By combining the predictions with SynGAP‐specific structural and clinical context, the SGM server provides a disease‐focused resource for examining effects of missense variation in SRD.

## 2. Results

### 2.1. Assessing the Accuracy of In Silico Missense Pathogenicity Predictors

To benchmark the performance of multiple in silico predictors, a test set was constructed from uncontested benign/likely benign and pathogenic/likely pathogenic ClinVar [3] entries (Figure 1d), together with selected variants from the conflicting category that showed a predominant benign or pathogenic classification trend. This curated dataset was used to evaluate the performance of in silico predictors in SRD detection. The ability of each method to classify variants as benign or pathogenic (Figure 2 and Tables 1 and S1) was assessed using bootstrap resampling (5000 iterations). In each iteration, variants were sampled with replacement from the original dataset, and performance metrics were recalculated. Reported values represent bootstrap means, and 95% confidence intervals (Table 1) and standard deviations (Table S1) were estimated from the bootstrap distributions.

**Figure 2:**
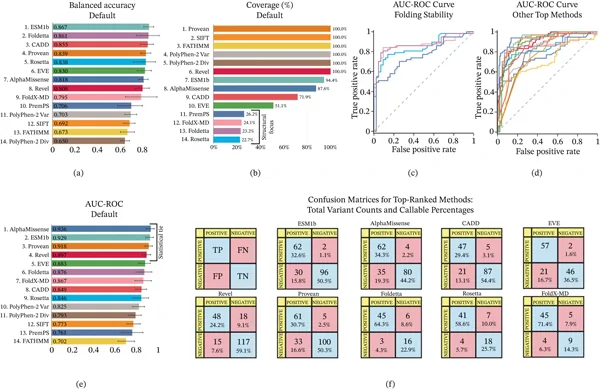
Benchmarking in silico predictors using clinical *SYNGAP1* missense variant data. (a) Balanced accuracy analysis identified ESM1b as the top‐performing method when using default classification thresholds; however, based on the overlapping confidence intervals, several other predictors, including Foldetta and CADD, performed comparably. When both balanced accuracy and (b) variant coverage were considered, PROVEAN and REVEL emerged as particularly effective predictors. In general, methods with lower balanced accuracy, such as SIFT or FATHMM, tended to achieve higher coverage, whereas folding‐stability methods with high balanced accuracy showed reduced coverage because they are restricted only to the structured N‐terminal PH‐C2‐GAP region. (c) ROC (receiver operating characteristic) curves indicated that PremPS was the weakest performing folding‐stability predictor, whereas Foldetta and FoldX‐MD performed comparably well. Among the other top‐performing methods, AlphaMissense appeared to provide the strongest early discrimination based on both (d) ROC curves and (e) AUC values; however, DeLong tests indicated performance comparable to that of ESM1b, PROVEAN, and REVEL. (f) Confusion matrices summarize true positive (TP), false positive (FP), true negative (TN), and false negative (FN) classifications. The matrices are shown as both variant counts and percentages, with percentages normalized separately for each method to sum to 100%, irrespective of the number of callable variants. Notably, the folding‐stability methods were characterized by particularly low FP and FN rates. All performance metrics were derived from bootstrap means (5000 iterations; 1000 for AUC).

**Table 1:** Performance of missense variant predictors on the clinical SynGAP variant dataset using default thresholds.

**Full SynGAP-*α*2 sequence (Res. 1–1343)**							
**Prediction method^a^**	**Recall^b^**	**Balanced accuracy^b^**	**F1 score^b^**	**MCC^b^**	**AUPRC** ^**b**^	**ROC-AUC** ^**c**^	**Coverage %** ^**d**^
AlphaMissense	0.939 (0.879–0.985)	0.818 (0.767–0.865)	0.762 (0.709–0.815)	0.615 (0.517–0.704)	0.917 (0.863‐0.962)	**0.937 (0.889–0.974)**	88
ESM1b	0.968 (0.921–1.000)	**0.866 (0.823–0.908)**	0.797 (0.745–0.853)	0.694 (0.611–0.777)	0.849 (0.762–0.925)	0.928 (0.890–0.961)	94
Provean	0.924 (0.864–0.985)	0.839 (0.793–0.887)	0.765 (0.709–0.821)	0.641 (0.552–0.732)	0.839 (0.759–0.909)	0.917 (0.878–0.951)	**100**
Revel	0.728 (0.621–0.833)	0.808 (0.749–0.867)	0.745 (0.667–0.827)	0.625 (0.510–0.742)	0.830 (0.758–0.896)	0.896 (0.850–0.937)	**100**
EVE	0.967 (0.914–1.000)	0.830 (0.763–0.888)	0.836 (0.778–0.891)	0.678 (0.553–0.785)	0.765 (0.655–0.883)	0.882 (0.824–0.932)	51
Foldetta	0.885 (0.788–0.962)	0.865 (0.759–0.953)	**0.910 (0.845–0.963)**	**0.700 (0.483–0.866)**	**0.926 (0.874–0.967)**	0.876 (0.803–0.944)	23
FoldX‐MD	0.901 (0.816–0.980)	0.799 (0.652–0.931)	**0.910 (0.846–0.962)**	0.579 (0.309–0.814)	0.922 (0.869–0.967)	0.867 (0.789–0.938)	24
CADD	0.903 (0.811–0.980)	0.855 (0.800–0.906)	0.784 (0.714–0.852)	0.674 (0.568–0.781)	0.753 (0.662–0.831)	0.849 (0.790–0.899)	72
Rosetta	0.856 (0.755–0.940)	0.838 (0.732–0.927)	0.883 (0.809–0.945)	0.656 (0.438–0.829)	0.902 (0.843–0.950)	0.846 (0.765–0.916)	23
PP‐2 Var	0.923 (0.848‐0.985)	0.703 (0.646–0.756)	0.619 (0.570–0.667)	0.400 (0.295–0.494)	0.758 (0.676–0.831)	0.825 (0.762–0.877)	**100**
PP‐2 Div	**0.985 (0.953–1.000)**	0.650 (0.609–0.692)	0.582 (0.550–0.614)	0.341 (0.265–0.406)	0.797 (0.722–0.862)	0.792 (0.730–0.848)	**100**
SIFT	0.881 (0.803–0.955)	0.692 (0.635–0.748)	0.612 (0.561–0.663)	0.375 (0.266–0.478)	0.808 (0.735–0.874)	0.771 (0.708–0.832)	**100**
PremPS	0.772 (0.660–0.882)	0.702 (0.585–0.806)	0.780 (0.690–0.864)	0.401 (0.168‐0.598)	0.807 (0.691–0.910)	0.759 (0.658–0.858)	27
FATHMM	0.607 (0.485–0.712)	0.672 (0.603–0.739)	0.568 (0.479–0.652)	0.336 (0.201–0.462)	0.600 (0.505–0.696)	0.701 (0.622–0.779)	**100**

**The PH-C2-GAP structural region of SynGAP1-*α*2 (Res. 198–730)**							
**Prediction method** ^**a**^	**Recall** ^**b**^	**Balanced accuracy** ^**b**^	**F1 score** ^**b**^	**MCC** ^**b**^	**AUPRC** ^**b**^	**ROC-AUC** ^**c**^	**Coverage %** ^**d**^
AlphaMissense	0.983 (0.947–1.000)	0.754 (0.675–0.829)	0.856 (0.814–0.898)	0.601 (0.457–0.732)	**0.962 (0.928–0.990)**	**0.953 (0.912–0.986)**	90
ESM1b	**1.000 (1.000–1.000)**	0.716 (0.634–0.800)	0.836 (0.797–0.880)	0.556 (0.420–0.686)	0.879 (0.797–0.953)	0.876 (0.800–0.939)	93
Provean	**1.000 (1.000–1.000)**	0.757 (0.686–0.837)	0.846 (0.808–0.891)	0.613 (0.502–0.736)	0.875 (0.795–0.943)	0.863 (0.781–0.934)	**100**
Revel	0.823 (0.719–0.912)	0.750 (0.665–0.831)	0.797 (0.726–0.867)	0.510 (0.347–0.676)	0.879 (0.814–0.937)	0.844 (0.768–0.914)	**100**
EVE	**1.000 (1.000–1.000)**	0.729 (0.630–0.833)	0.891 (0.846–0.932)	0.604 (0.442–0.759)	0.791 (0.672–0.913)	0.833 (0.728–0.919)	70
Foldetta	0.883 (0.792–0.962)	**0.865 (0.767–0.950)**	0.910 (0.849–0.962)	**0.700 (0.523–0.869)**	0.926 (0.880–0.965)	0.877 (0.803–0.939)	59
FoldX‐MD	0.902 (0.816‐0.979)	0.800 (0.658–0.925)	**0.911 (0.851–0.962)**	0.585 (0.317–0.815)	0.922 (0.870–0.964)	0.867 (0.790–0.938)	61
CADD	0.936 (0.854–1.000)	0.768 (0.671–0.864)	0.851 (0.785–0.913)	0.587 (0.405–0.764)	0.803 (0.716–0.886)	0.751 (0.641–0.853)	67
Rosetta	0.854 (0.750–0.954)	0.839 (0.740–0.926)	0.882 (0.814–0.947)	0.657 (0.471–0.829)	0.902 (0.844–0.949)	0.847 (0.767–0.917)	57
PP‐2 Var	0.947 (0.877–1.000)	0.647 (0.570–0.726)	0.774 (0.726–0.818)	0.379 (0.209–0.533)	0.859 (0.792–0.919)	0.786 (0.692–0.870)	**100**
PP‐2 Div	**1.000 (1.000–1.000)**	0.626 (0.558–0.698)	0.777 (0.748–0.812)	0.398 (0.264–0.520)	0.884 (0.826–0.932)	0.677 (0.577–0.776)	**100**
SIFT	0.911 (0.825–0.982)	0.711 (0.619–0.793)	0.800 (0.742–0.853)	0.472 (0.277–0.633)	0.922 (0.878–0.959)	0.835 (0.749–0.908)	**100**
PremPS	0.770 (0.646–0.886)	0.700 (0.593–0.808)	0.778 (0.689–0.860)	0.399 (0.185–0.609)	0.805 (0.705–0.898)	0.758 (0.667–0.845)	68
FATHMM	0.634 (0.509–0.754)	0.561 (0.463–0.656)	0.627 (0.527–0.715)	0.123 (−0.073 to 0.315)	0.706 (0.607–0.796)	0.636 (0.520–0.745)	**100**

*Note:* For folding stability methods (FoldX‐MD, Rosetta, Foldetta, and PremPS), predictions were restricted to the N‐terminal SynGAP structural model (Residues 198–730; Figure 1b). Consequently, their performance metrics are directly comparable only within this subset, which comprises ~30% of the SynGAP missense variants in the SGM database. Values are reported as bootstrapped means ± confidence intervals. The bootstrapped balanced accuracy estimates and DeLong significance tests for ROC‐AUC comparisons are also presented in Figures 1 and S2. The same metrics are given with standard deviation (SD) in Table S1.

^a^Prediction methods: Methods evaluated include AlphaMissense [42], ESM1b [41], PolyPhen‐2 (HumDiv and HumVar) [47], FATHMM [48] SIFT [39], PROVEAN [49], REVEL [40], CADD [50], EVE [51], FoldX‐MD [52, 53], Rosetta [54, 55], Foldetta [56], and PremPS [57]. Pathogenic and likely pathogenic ClinVar variants were combined and treated as positive cases, whereas benign and likely benign variants were combined and treated as negative cases. The best performing method for each metric, based only on the bootstrapped mean value, is bolded.

^b^Threshold‐dependent performance metrics: recall (sensitivity), balanced accuracy, F1 score, Matthews correlation coefficient (MCC), and area under the precision‐recall curve (AUPRC) were calculated using the default classification thresholds provided by each method. Definitions of all metrics are provided in Eqs. 1‐7 of the SI. The default thresholds are given in Table S2.

^c^ROC‐AUC: The area under the receiver operating characteristic curve (ROC‐AUC) evaluates the ability of a method to discriminate between pathogenic and benign variants across all possible classification thresholds and is therefore independent of the default threshold used for classification.

^d^Coverage %: Coverage or prediction rate denotes the proportion of SynGAP missense variants for which a method generated predictions. Rates are reported both for the entire SynGAP protein (Residues 1–1343) and for the N‐terminal structural region (Residues 198–730).

Multiple complementary performance metrics were evaluated (Table 1). Balanced accuracy and the Matthews correlation coefficient (MCC) are used as the primary measures of classification performance, whereas the area under the receiver operating characteristic curve (ROC‐AUC), area under the precision‐recall curve (AUPRC), recall, and F1 score are providing additional insight into discriminatory power and threshold‐dependent behavior. The mathematical formulations underlying these analyses are provided in the SI (Eqs. 1–9), together with methodological descriptions. Substantial differences were observed between methods, with folding stability‐based approaches and DL‐based predictors demonstrating particularly strong performance (Figure 2a–f).

Folding stability calculations that focus on missense variants within the structured PH‐C2‐GAP region provided highly accurate predictions (Figure 2a and Table 1). Foldetta [56], which integrates predictions from both Rosetta [54, 58, 59] and FoldX‐MD [38, 52, 53], demonstrated excellent overall performance, achieving among the highest values for balanced accuracy (0.865), MCC (0.700), and AUPRC (0.926) (Figure 2a,b and Table 1). Rosetta [59, 60] also performed strongly and, across some evaluated metrics, slightly outperformed FoldX‐MD [38, 52]. However, several non‐folding stability‐based predictors, including ESM1b, PROVEAN [61], AlphaMissense [42], and EVE [51], generated similarly high‐quality predictions, as indicated by substantial overlap in the bootstrap confidence intervals (Figure 2a,b and Table 1).

The ESM1b [41] language model showed particularly strong overall performance, achieving the highest balanced accuracy (0.866) while providing predictions beyond currently resolved structural regions and covering 94% of variants included in the SGM server database (Figure 2 and Table 1). Likewise, PROVEAN [61] and AlphaMissense [42] produced robust predictions with high balanced accuracy values. Notably, AlphaMissense [42] achieved the highest ROC‐AUC (0.937) among all methods evaluated, indicating excellent discrimination between benign and pathogenic variants across classification thresholds (Figure 2d,e and Table 1). In contrast, SIFT [39], PolyPhen‐2 [47], and FATHMM [48] displayed lower balanced accuracy and MCC values, indicating reduced classification performance (Figure 2a and Table 1).

Using a baseline AUPRC of 0.33 as a reference, all methods demonstrated better‐than‐baseline performance in identifying pathogenic variants, with AUPRC values ranging from 0.600 to 0.926 (Table 1). Folding stability methods and AlphaMissense achieved excellent performance, with AUPRC values exceeding 0.9. ESM1b, PROVEAN, and REVEL also showed strong performance, with AUPRC values above 0.8.

When the analysis was restricted to missense variants only within the PH‐C2‐GAP structural region for all methods, folding stability calculations were clearly the strongest predictors (Figure S2a,b and Table 1). Foldetta [56] achieved the highest MCC (0.700) and one of the highest AUPRC values (0.926), whereas FoldX‐MD [52] and Rosetta [59] also demonstrated strong performance individually. Foldetta [56] further achieved the highest balanced accuracy (0.865) and among the highest recall values, while nearly sharing the highest F1 score with FoldX‐MD (0.910 vs. 0.911). Notably, AlphaMissense [42], ESM1b [41], EVE [51], PROVEAN [61], PolyPhen‐2 [47], and SIFT [39] exhibited higher recall values, suggesting a greater tendency to classify variants as pathogenic, accompanied by reduced specificity.

However, AlphaMissense [42] achieved the highest ROC‐AUC even with this structural focus (0.953; Figure S2c‐d; Table 1), indicating strong discriminatory power and suggesting that threshold optimization could further improve its classification performance. Collectively, these results highlight the value of folding stability calculations when reliable structural template models are available. In their absence, or when stability predictions are uncertain, AlphaMissense [42], ESM1b [41], and PROVEAN [61] under default cutoffs provide highly effective alternatives with substantially broader coverage.

### 2.2. Threshold Optimization Yields the Largest Gains for AlphaMissense

The ROC‐AUC values and ROC curves (Figures 2c–e and S2c,d and Tables 1 and S1) indicated that several predictors possessed discriminatory power that was not fully reflected by their default classification thresholds. Among the evaluated methods, AlphaMissense [42] emerged as the strongest candidate for threshold optimization, as it achieved the highest ROC‐AUC value while exhibiting only midtier balanced accuracy and MCC under its default classification scheme. Although AlphaMissense [42] produced the highest ROC‐AUC estimate, DeLong testing showed that its performance was actually statistically indistinguishable from that of ESM1b [41], PROVEAN [61], and REVEL [40] (Figure 2e).

To investigate this discrepancy, prediction thresholds were systematically optimized for all predictor methods by evaluating score‐based cutoffs and selecting values that maximized Youden′s J statistic [62], which is essentially equivalent to maximizing balanced accuracy (Figure 3). Under a single‐threshold framework, optimization removed the intermediate classification category and substantially increased variant coverage (Figure 3a,b and Table S2). AlphaMissense [42] exhibited the largest performance improvement, with balanced accuracy increasing from 0.82 to 0.90 and MCC from 0.61 to 0.77 (Figures 2a and 3a and Table S3). Several other predictors, including REVEL [40] and PolyPhen‐2 [47], also benefited from threshold optimization. When focusing on MCC, optimization improved the performance of FoldX‐MD and Foldetta, whereas Rosetta exhibited a modest decline. Notably, all three folding stability methods achieved substantially greater variant coverage following threshold optimization, increasing from ~23%–27% to as high as 40% (Figures 2b and 3b and Table S3). However, because folding stability represents a biophysical property rather than a direct measure of pathogenicity, the biological relevance of this kind of optimized classification schemes remains uncertain.

**Figure 3:**
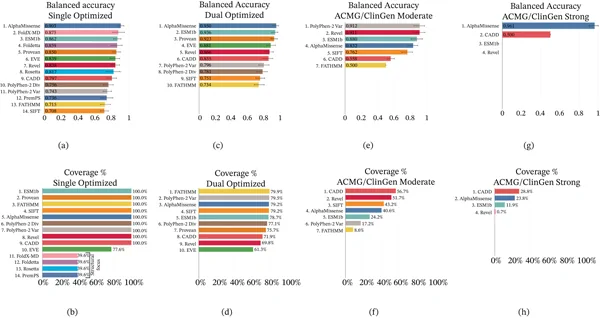
Comparison of balanced accuracy and coverage for *SYNGAP1*‐ and ACMG/ClinGen–calibrated pathogenicity prediction methods. Single‐threshold optimization improved both (a) balanced accuracy and (b) variant coverage relative to the default threshold settings (Figure 2a,b). Dual‐threshold optimization, which classifies variants as benign, uncertain, or pathogenic, produced (c) further gains in balanced accuracy, whereas (d) modestly reducing overall coverage across variants included in the SGM server. Under ACMG/ClinGen–derived thresholds, the moderate framework achieved high balanced accuracy for methods with published global calibration ranges [63, 64], but at the cost of substantially reduced coverage compared with the default (Figure 2), single‐threshold, and dual‐threshold approaches. The more stringent ACMG/ClinGen strong framework further increased (g) balanced accuracy for AlphaMissense while even reducing performance for CADD, but, at the same time, (h) severely reduced variant coverage (see Figure 2 for further interpretation).

Dual‐threshold optimization, which classifies variants as benign, uncertain, or pathogenic, further improved the performance of several in silico predictors (Figure 3c,d). Although most methods benefited from this approach (folding‐stability methods excluded from this analysis), AlphaMissense [42] again demonstrated the largest absolute improvement. Relative to both its default classification scheme (Figure 2a) and the optimized single‐threshold implementation (Figure 3a), dual‐threshold optimization substantially increased balanced accuracy and MCC (Figure 3c and Table S3). REVEL [40] likewise showed measurable gains following gene‐specific optimization. Because CADD [50] lacks established default pathogenicity thresholds, optimization is required before any kind of variant classification can be performed; notably, the dual‐threshold framework consistently outperformed the more unconventional single‐threshold approach in balanced accuracy (Figure 3a,c and Table S3). Collectively, these results support the value of *SYNGAP1* gene‐specific threshold calibration for missense pathogenicity predictors that produce continuous score distributions.

For AlphaMissense [42], stratified 5‐fold cross‐validation demonstrated that thresholds optimized using the full benchmark dataset generalized well across validation folds (Figure S3), yielding a mean test balanced accuracy of 0.904 compared with 0.901 for the training set. Optimized CADD [50] thresholds also showed reasonable generalization, although with greater variability (Figure S3). In contrast, fold‐specific optimization was less stable, likely owing to the smaller training sets obtained after subdivision of the already limited variant dataset (not shown). Nevertheless, the median optimized thresholds and prediction coverages remained highly similar across folds for both AlphaMissense [42] and CADD [50], supporting the suitability of the optimized cutoffs for classification use.

Owing to its broad variant coverage, continuous score distribution, and substantial performance gains following threshold optimization, the optimized AlphaMissense classification scheme was incorporated into the SGM server as “AlphaMissense: Optimized”. The optimized CADD [50] thresholds were simply implemented as “CADD”. Although optimization also improved the performance of several other predictors, including REVEL [40], the magnitude and consistency of these gains were generally less compelling in validation (not shown). Mainly, the increased fold‐to‐fold variability observed for these other methods suggests a potential for overfitting. Consequently, optimized threshold schemes were not adopted more broadly, but as additional clinically assigned variants become available, reevaluation of their calibration should be done.

### 2.3. ACMG/ClinGen Thresholds Severely Reduce Prediction Coverage

ACMG/ClinGen–calibrated prediction thresholds were developed as broadly applicable frameworks for variant interpretation across many genes [43]. Among the predictors evaluated here, REVEL [40], AlphaMissense [42], and ESM1b [41] include calibrated score ranges capable of reaching PP3_Strong evidence levels, whereas FATHMM [48], PolyPhen‐2 [47], SIFT [39], and CADD [50] contribute through PP3/BP4–calibrated thresholds [63, 64]. These frameworks aim to minimize false pathogenic and false benign classifications while supporting clinical variant interpretation and were not intended for standalone predictor benchmarking. Nevertheless, for comparison, two ACMG‐based threshold configurations were devised: (1) a moderate setting and (2) a more stringent strong setting.

The ACMG moderate thresholds generally achieved performance comparable to, or better than, the default thresholds of individual methods (Figure 3e,f). The ACMG strong thresholds often yielded the highest predictive accuracy (Figure 3g,h), and for AlphaMissense [42] and PolyPhen‐2 [47] even exceeded the performance obtained through *SYNGAP1*‐specific threshold optimization. These gains came at the cost of substantially reduced coverage, as far fewer variants met the classification criteria (Figure 3f,h). Under the ACMG strong framework, REVEL [40] classified only 0.7% of variants, whereas AlphaMissense [42] coverage declined to 23.8%. Although the moderate thresholds improved coverage, most predictors still classified considerably fewer variants than under optimized settings. This effect was particularly pronounced for FATHMM [48], whereas REVEL [40] and AlphaMissense [42] retained more practical levels of applicability.

Overall, the results suggest that the ACMG/ClinGen ranges may be overly stringent for *SYNGAP1* missense variant interpretation. Although they often offer high predictive accuracy, they do so at the expense of variant coverage. In contrast, *SYNGAP1*‐specific calibration provides a far more favorable balance between accuracy and applicability.

### 2.4. Shared Pathogenicity Signals Across Prediction Methods

Variant‐by‐variant comparisons (Tables S4–S5 and Figure S4) showed positive phi (*ϕ*) coefficients for all predictor pairs (0.05–1), indicating varying degrees of agreement between methods. The *φ* is a statistical measure used to calculate the strength and direction of association between binary classifications, with higher positive values indicating stronger agreement among predictors. Among sequence‐based predictors, AlphaMissense [42] showed particularly strong agreement with EVE [51] (0.77), ESM1b [41] (0.67), and PROVEAN [61] (0.64; Table S4). Similarly, FoldX‐MD [38, 52] and Rosetta [58] were highly concordant (0.89; Table S5), supporting their combined implementation in Foldetta [56] (Figure 2 and Table 1).

Correlation analysis of prediction scores yielded similar observations. The *R* ^2^ scores AlphaMissense [42] and ESM1b [41] were moderately correlated (0.48; Figure S4a), whereas the relationship between ESM1b [41] and FoldX‐MD [38, 52] was weaker (0.23; Figure S4b). Scatter plots and Venn diagrams (Figure S4a–c) demonstrated substantial overlap among DL‐based predictors, suggesting that they frequently capture similar pathogenicity signals. In contrast, overlap between FoldX‐MD [38, 52] and nonstructural predictors was more limited (Figure S4c–e), reflecting the distinct structural focus of the folding stability analyses.

Overall, despite methodological differences, the predictors appeared to capture a largely shared pathogenicity signal. The substantial agreement among the top‐performing methods suggested potentially useful gains from applying a consensus scoring approach. Consequently, a 3‐out‐of‐4 majority‐vote or consensus scheme (ESM1b [41], AlphaMissense [42], REVEL [40], PROVEAN [61]) was implemented in the SGM server to provide a conservative summary of predictor agreement using default thresholds. However, as optimized AlphaMissense performed better than the consensus approach in variant classification, the latter is not considered further here.

### 2.5. Structural Domains Are Highly Enriched With Pathogenic Missense Variants

As robust predictor performance was achieved via AlphaMissense threshold optimization, a detailed look was next taken into its predictions. This was motivated by the method′s high accuracy in classifying missense variants both here (Figure 2 and Table 1) and in other benchmarking studies [37, 42]. When analyzing the scores and default classifications, particular attention was paid to pathogenic variants′ structural domain distribution or pathogenic susceptibility, performance across different sets, and differences between predicted ordered and disordered regions.

AlphaMissense predictions across domains and motifs (Figures 4a,b and S5) revealed structural differences in susceptibility to pathogenic missense variation. In ClinVar, the GAP (*N* = 33) and C2 (*N* = 18) domains contain the majority of pathogenic missense variants, far exceeding nondomain regions (*N* = 8). However, VUS dominate overall (*N* = 618), limiting interpretation (Figure S5a). Predictions indicated that nondomain regions account for 50.5% of all variants but only 16.8% are pathogenic compared with 34% in domain regions (Figure S5c). In contrast, despite their smaller size, the PH (~78%), C2 (59%), GAP (~75%), and CC (~70%) domains are highly enriched for pathogenic variants. Notably, the small NPxF motif (~93%) was predicted to be almost entirely pathogenic (Figure 4b and Table S6). Structured domains exhibit similar pathogenic density (~3.4–4.6 per residue), but NPxF stands out (5.7), whereas the putative SH3‐binding motif shows a much lower density (1.9; Figure S5a‐b; Table S6).

**Figure 4:**
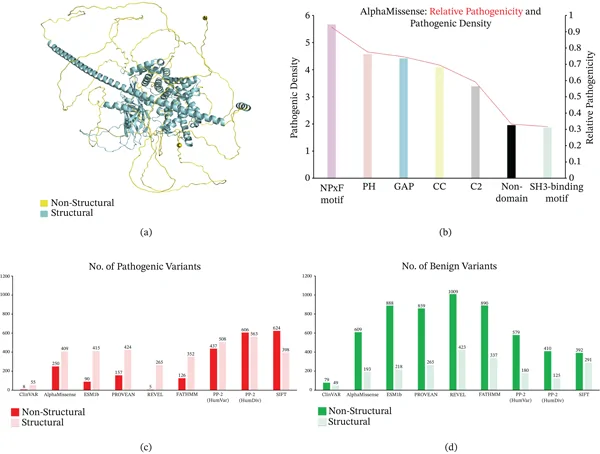
Structural distribution and pathogenicity of predictions for SynGAP missense variants. (a) The AlphaFold model of SynGAP1 monomer (AF‐Q96PV0‐F1‐v4, cartoon representation) shows structured regions in cyan and disordered or non‐structured regions in yellow. (b) Relative pathogenicity (pathogenic variants per domain length) and pathogenic density (pathogenic variants per total domain variant count) identify the NPxF motif as a concentrated hotspot based on AlphaMissense pathogenicity predictions. The PH, GAP, and CC domains also show notable enrichment. Due to perfect prediction coverage rate for all potential variants, these domain/motif‐based results for AlphaMissense are very significant based on Fisher′s exact tests. (c) Pathogenic missense variants are predominantly predicted to occur in structured regions, particularly within the N‐terminal model (Res. 198–730) and the CC domain (1170–1270). (d) In contrast, benign variants are mostly predicted to occur in disordered regions (1–197, 731–1169, and 1271–1342). Note that NPxF motif and putative SH3 binding motif are not considered here as domains.

AlphaMissense average scores (Table S7) suggested that the domain/motif regions (~0.7) were more pathogenic than the nondomain areas (0.42). NPxF ranked the highest (0.89), followed by PH, GAP, CC, and C2 (0.79–0.62), whereas SH3‐binding motif remained the lowest (0.41). When restricting analysis to variants categorized as pathogenic, scores rose markedly, but pathogenicity ranking of domains remained unchanged. ClinVar‐listed variants had higher scores than the gnomAD‐listed ones (0.51 vs. 0.41; Table S7), as expected since gnomAD includes general population variants. Theoretical variants (not in ClinVar or gnomAD) show the highest average score (0.59), suggesting yet undiscovered pathogenic variants may remain. For domains such as C2 (0.91 vs. 0.90), GAP (0.92 vs. 0.90), and SH3‐binding motif (0.78 vs. 0.76), ClinVar‐listed pathogenic variants score even higher than theoretical ones, indicating that the severely pathogenic variants were enriched in the patient data. The gnomAD variants vary by rarity: allele counts of 1–10 average 0.43, dropping to 0.31 for 11–100 and 0.14 for > 100, reflecting the inverse relationship between population frequency and predicted pathogenicity (Table S8).

Lastly, the AlphaMissense scoring on the SynGAP′s predicted ordered and disordered regions was analyzed. Different in silico methods assessing disorder agree largely on structural segmentation of SynGAP (Figure S1). Based on IUPred ordered/disordered division [65], AlphaMissense assigns more pathogenic variants to the ordered regions (32.5%) and more benign variants to disordered regions (18.4%; Figure S5c,d). Despite SynGAP being largely intrinsically disordered (Figure S1), pathogenic predictions dominate ordered segments (*N* = 2624 vs. 1217 benign/unclear), whereas IDRs house more benign predictions (*N* = 2162 vs. 2092 pathogenic/unclear). Prediction strength is also significantly higher in ordered regions (0.70 vs. 0.42; Table S7), indicating stronger prediction confidence in structured segments. Importantly, this division inserting more often pathogenic variants on structured domain regions (Figure 4c) and benign variants in the nonstructured regions (Figure 4d) is visible in the ClinVar data and all pathogenicity prediction methods tested.

### 2.6. Missense Variation Could Affect Phase Separation and Phosphoregulation

Although SynGAP‐IDR variants are typically predicted to be benign for example by AlphaMissense (Figure S5c), they could still exert pathogenic effects by evoking abnormal phase separation (PS) behavior.

Firstly, the potential effects of missense variants on PS were explored computationally. PSMutPred [66] identified 70 missense variants as PS‐impacting (IP RF; threshold > 0.5; Figure 5a). Among ClinVar variants, only three VUS met the criterion (R73K, K108R, and C282S). The PS‐impact scores averaged at 0.35 for all variants and 0.53 for impacted ones, maxing out at 0.64. The impact variants were mostly predicted to weaken the PS (*N* = 59; SP RF < 0.5) rather than strengthen it (*N* = 11; SP RF > 0.5) and out of the Top 20 most impactful variants, only three were predicted to strengthen PS (Figure 5b). Most PS‐impact variants occurred in nondomain regions (*N* = 41; Table S9), with fewer in C2 (*N* = 14), GAP (*N* = 5), CC (*N* = 4), PH (*N* = 2), and the SH3‐binding motif (*N* = 4). This enrichment in nondomain regions aligns with the established role of IDRs in promoting PS [68]. When grouped by a 25‐aa proximity rule (Table S10), the largest clusters occurred at the N‐terminus (*N* = 16 for Residues 100–185; *N* = 8 for 3–73), C2 domain (*N* = 8 for 362–397; *N* = 6 for 282‐331), and putative SH3‐binding motif (*N* = 12 for 752–821).

**Figure 5:**
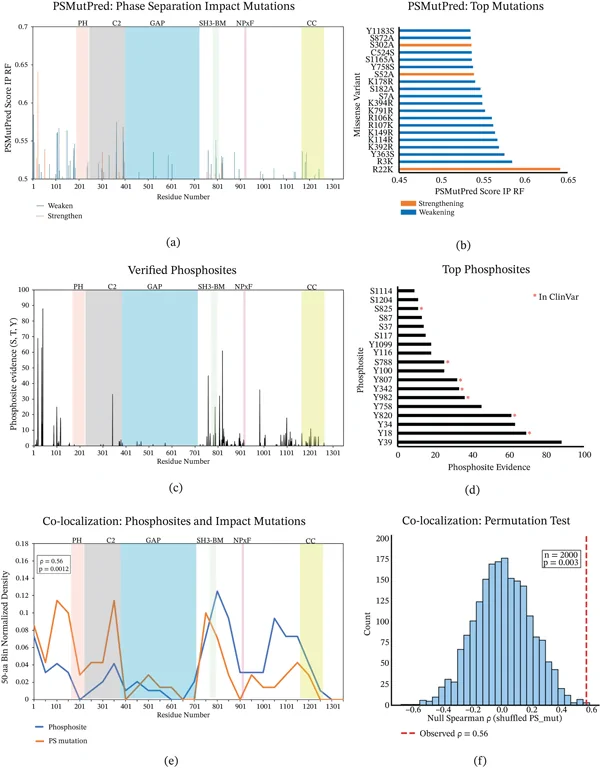
Phase separation and phosphoregulation landscape of SynGAP. (a) Missense variants predicted by PSMutPred [66] to impact PS (IP RF > 0.5 threshold) are plotted along the SynGAP sequence using their IP RF scores. Variants predicted to weaken PS are shown in blue, whereas those predicted to strengthen it are shown in orange, based on SP RF scores (> 0.5 threshold). For each residue, only the missense variant with the highest IP RF score is displayed. (b) Using the same color scheme, the top‐impact missense variants (ranked by IP RF score) are shown. (c) Experimentally validated phosphosites (T, S, and Y; Table S13), obtained from PhosphoSitePlus [67], are plotted based on the amount of supporting evidence. (d) The most strongly supported phosphosites are shown. (e) Colocalization or coclustering analysis of phosphosites (blue line) and predicted phase separation‐impacting variants (orange line) is shown using a sliding window of 50 amino acids summed variant or phosphosite densities along the sequence. (f) Permutation test (2000 random shuffles of variant positions) showed that only 0.3% of shuffled datasets produced Spearman correlation as strong as the observed correlation between predicted PS impact variants and verified phosphosite positions (*ρ* = 0.56).

Secondly, missense changes may also disrupt posttranslational modifications (PTMs) such as phosphosites, thereby affecting their normal role in regulating LLPS. To investigate this, experimentally verified SynGAP phosphosites were examined in detail (Figure 5c and Tables S11–S12). These sites were mostly clustered outside the PH‐C2‐GAP region based on available experimental data [67]; however, the NPxF motif and CC domain overlapped with notable phosphosite clusters. Only four highly evidenced sites (Figure 5d and Table S12) were located within the C2 domain (Y342), SH3‐binding motif (S788; Y807), and CC domain (S1204), compared with 14 sites in nondomain regions. Four major phosphosite clusters were identified using the ≤ 25‐aa proximity rule (Table S11), spanning residue ranges 7–39, 85–117, 723–913, and 1053–1261. Notably, actual kinase phosphorylation motifs are shorter [69] and applying a stricter proximity rule would generate a more scattered phosphosite landscape. In total, 56 ClinVar variants, mostly with unclear or benign status, overlapped with verified phosphosites, except for S842Y, which is classified as likely pathogenic.

Thirdly, in close inspection, it was found that phosphosites and predicted PS‐impact sites clearly coincide in the SynGAP sequence when their occurrences were plotted against SynGAP‐*α*2 sequence (Figure 5a vs. Figure 5c). When both variables were aggregated into 50‐aa density bins, this colocalization became even more evident (Figure 5e). Specifically, the two distributions exhibit a clear monotonic relationship, meaning they tend to increase and decrease together and, importantly, chi squared test (*χ* ^2^ = 27.85, *p* = 0.006; bin = 100) and Spearman correlation coefficient (*ρ* = 0.56; bin = 50) indicate a statistically significant moderate positive association. Permutation test (Figure 5f) of 2000 random shuffles demonstrated that only 0.3% (*p* = 0.003) of shuffles achieved similar or stronger correlation compared with the observed correlation, which indicated that the colocalization was unlikely to be random. There was also a moderate correlation between phosphoresidues (S, T, and Y) and PS‐impacting variants (*ρ* = 0.45; bin = 50) coclustering, whereas the correlation between phosphoresidues and verified phosphosites was logically even higher (*ρ* = 0.57; bin = 50). Notably, 19% of PS‐impacting variants directly affected residues that match the known phosphosites, and the number would undoubtedly be higher if considering the phosphorylation recognition motif residues as well.

These findings suggest that phosphosites and PS‐impacting sites may together form phase transition prone regions. This could be important not only for normal SynGAP function, but it also provides a plausible disease mechanism by which at least some pathogenic variants could alter PS.

### 2.7. SGM Server: An Online Platform for Assessing SynGAP1 Missense Variant Pathogenicity

To support effective missense variant assessment and classification, the SGM server (Figure 6) was developed. The server functions as an online portal and data repository for assessing SynGAP missense variant pathogenicity using linked online database information and predictions from top‐performing in silico methods (Figure 1f). The user interface is designed to be fast, searchable, and easy to use, enabling evaluation of missense effects both structurally and through numerical or categorical outputs. Predictions are available not only for ClinVar‐ and gnomAD‐listed variants but also for all potential variants arising from single‐base substitutions. This functionality extends the utility of the framework beyond the current clinical datasets, facilitating the evaluation of newly reported *SYNGAP1* variants as additional evidence accumulates.

**Figure 6:**
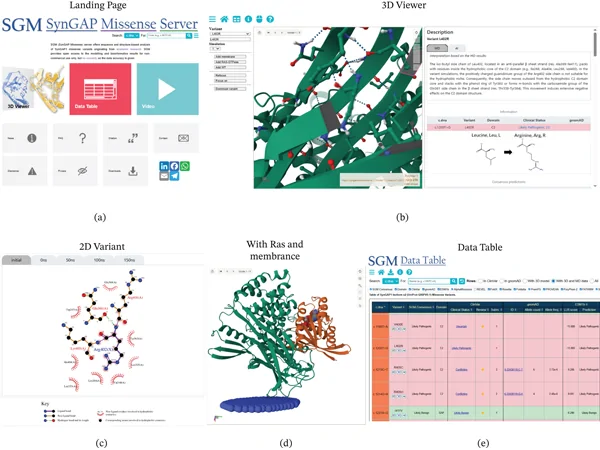
SynGAP Missense server. (a) The SGM server landing page presents three main sections: *3D Viewer*, *Video*, and *Data Table*, and several other subsections including *Privacy*, *FAQ*, and *Downloads*. (b) The MOL∗‐based [70] *3D Viewer* allows the user to examine the SynGAP missense variants in 3D. (c) The interactions of the mutated residues with nearby residues can also be inspected via 2D diagrams [71]. (d) The interface includes specifically built‐in buttons for focusing or refocusing on specific residues as well as visualizing them against WT‐SynGAP or in complex with membrane and/or RasGTPase (PDB: 1WQ1) [72]. (e) The *Data Table* includes existing clinical data on the missense variants, pathogenicity predictions, and MD simulation metrics. It is fully searchable, sortable, and downloadable and includes show/hide and radio buttons for rearranging the data.

The server features a MOL ^∗^‐based [70] *3D Viewer* (Figure 6b–d) with 2D interaction diagrams for visualizing missense variants in the protein′s PH‐C2‐GAP region (Figure 6c). For 211 variants previously analyzed through atomistic molecular dynamics (MD) simulations [46], users can inspect trajectory snapshots, various analysis plots (e.g., RMSD; Figure S6), and written MD summaries (Figure 6b). The *Data Table* section (Figure 6e) combines sequence‐, structure‐, and MD‐based analyses with linked variant data and pathogenicity predictions from sequence‐ and/or structure‐based methods, folding stability calculations, machine learning (ML)‐ and DL‐based approaches. Additional metrics such as MW*Δ*, hydropathy *Δ*, evolutionary metrics (e.g., PAM120) [73], and 2D substitution diagrams are included. The table is fully searchable, sortable, downloadable, and predictions for each variant are summarized using AI for improved interpretability. The same variant‐specific information shown collectively in the *Data Table* is also available individually for each variant next to the 3D Canvas window of MOL ^∗^ [70] in the *3D Viewer*.

## 3. Discussion

It is vital to fast‐track the diagnosis of de novo missense variants in SRD patients. VUS occur at ~1.5 times the frequency of those with definitive annotations, highlighting the fact that diagnostic interpretation is lagging far behind variant identification (Figure 1d,f). This annotation gap was previously addressed using folding stability‐based predictions and atomistic MD simulations that evaluated the structural and dynamic consequences of a large subset of established variants [46]. This structural focus was justified, as folding stability calculations deliver the best accuracy without excessive threshold adjustment (Figure 2 and Table 1). This is logical because the impact of any missense variant depends on its specific effect on the protein structure or folding dynamics (Figure 1b,e), and without structural context, reliable annotation is difficult [2, 33, 34, 74–76].

While assuring high accuracy and allowing visual interpretation, this structural focus is also a tangible limitation since roughly half of the SynGAP sequence is predicted as IDR, lacking permanent secondary structure (Figures 4a and S1). Although folding stability calculations were expanded to all likely variants for the PH‐C2‐GAP region (*N* = 3476), 4475 potential variants, including 415 ClinVar variants, still lie completely outside the structured regions. To address this, a wide variety of different sequence‐based or hybrid sequence/structure‐based pathogenicity predictors were explored (Figures 2 and S7 and Table 1) and assessed to determine whether any individual method (Table 1) could maximize coverage and accuracy for potential diagnostic purposes. Benchmarking shows that the latest DL methods, ESM1b and AlphaMissense, perform excellently, with the latter method particularly strong after threshold optimization (Figures 2a and 3c and Table S3). Notably, despite the optimization boost in AlphaMissense [42] performance, the DeLong test [77], which is based on AUC calculations and, therefore, independent of threshold selection, indicates its performance is statistically comparable to that of ESM1b [41], PROVEAN [61], and REVEL [40].

Although pathogenicity predictions can be acquired for variants outside the established domains of SynGAP using top‐performing predictors (Figure 1f), the benchmarking reveals a potential problem. The testing relies on ClinVar data (Figure 1d), which contains far more pathogenic than benign (Figure 4c vs. Figure 4d) annotations for missense variants in structural regions (Figure S5a). This bias or preference may stem from clinical annotation processes, where variants in functional domains are likely more readily classified as pathogenic. The same bias is also observed in the predictions (Figure 4c,d) from sequence‐based methods like PROVEAN and DL‐based approaches, regardless of structural input. This trend, which has been noted in larger‐scale benchmarking studies, may reflect a broader limitation in assessing IDR variants [78]. Hence, although this pattern could indicate true biological missense susceptibility in structured regions, it may also highlight predictor limitations in poorly conserved or structurally unresolved regions [37, 46, 66, 78].

Human annotation can introduce bias when clinically curated datasets are used for model training, as seen for predictors such as PolyPhen‐2 and FATHMM [47, 48, 79]. In contrast, ESM1b and EVE, and to a lesser extent AlphaMissense, mitigate this circularity because their underlying models are not directly trained on clinical pathogenicity labels, although AlphaMissense incorporates labeled variant data during score calibration [41, 42, 51, 79]. AlphaMissense also integrates AlphaFold 3D models for structural context and performs better in structured regions than in disordered ones [37, 42], though accuracy declines in low‐quality templates or membrane‐interacting disordered segments [37]. Even ESM1b, despite lacking explicit structural input, appears to possess a rudimentary understanding of structure or at least domain organization [41]. Therefore, adjusting AlphaMissense thresholds using human‐annotated sets (Figure 1d) may further compromise classification performance, a worry that compounds concerns about overfitting given the limited set size. It is worth noting that the optimized AlphaMissense thresholds, which will need to be reevaluated as more data become available, are broadly consistent with the values reported for strongly pathogenic threshold intervals (PP3+3/+4) in Bergquist et al. [64]. Nevertheless, rigorous testing with any such sets (Figure 2 and Table 1) cannot guarantee prediction accuracy (Figure 1f). To date, Meili et al. provide the only systematic experimental assessment of SynGAP missense variant effects (*N* = 57) through protein stability measurements [15]. These kinds of experimentally derived datasets may ultimately provide an objective framework for benchmarking, calibrating, and refining variant effect predictors, while reducing risks associated with circularity and overfitting.

Regardless, a major advantage of the gene‐specific calibration strategy is the incorporation of a minimum 80% coverage requirement during threshold optimization. This approach preserves broad applicability while maintaining predictive performance comparable to that achieved by the ACMG strong thresholds (Figure 3 and Table S3). In practical settings, providing a reliable classification for a large proportion of variants is often as important as maximizing prediction confidence, particularly because many variants do not reach the stringent score ranges required for ACMG strong evidence. Consequently, the optimized thresholds appear to offer a more favorable balance between accuracy and coverage than either the default or ACMG strong/moderate thresholds, potentially making them better suited for routine assessment of SynGAP variants. Still, when maximal confidence is required and reduced coverage is acceptable, ACMG strong (e.g., AlphaMissense) and moderate (e.g., REVEL) thresholds are reliable conservative in silico options. To our knowledge, ACMG evidence‐strength thresholds have not been calibrated for *SYNGAP1* yet and it is an open question if the framework works for a gene with this high level of burden of pathogenic missense variation.

Despite having generally lower AlphaMissense scores and fewer known pathogenic entries in the disordered regions compared with its structured domains (Figure S5c and Table S7), the SynGAP‐IDRs are unlikely to be completely immune to missense variation. Indeed, this is expected as AlphaMissense is known to exhibit greater prediction uncertainty for variants within IDRs than those in the structured domains [66]. Furthermore, ANCHOR [80] predicts extensive regions of disordered binding propensity across IDRs (Figure S1e; > 0.5) and in the N‐terminal segment (Residues 81–268), regions flanking the putative SH3‐binding motif (742–790, 799–852, and 860–891), the section beginning with the NPxF motif (902–1186), and the C‐terminal section (1258–1343). These regions may mediate transient protein‐protein interactions, similar to the established role of the NPxF motif (924‐932) complexing for example with AIDA‐1 [9]. Missense variants within these transient motifs could disrupt, weaken, or enhance PSD scaffolding interactions that are critical for LLPS, potentially resulting in gain or loss of essential functions. Supporting this, PSMutPred [66] predictions suggest that missense variation within IDRs may modulate LLPS, typically by weakening phase separation propensity (Figure 5a,b). Additionally, variants at experimentally validated phosphosites could interfere with phosphoregulation, potentially preventing the toggling of LLPS activity on or off (Figure 5c,d).

Notably, both phosphosites and predicted LLPS‐impact variants show high level of colocalization within the SynGAP‐IDRs (Figure 5e). In other words, predicted PS‐impacted areas and phosphosites colocalize (Figure 5e) in the protein′s flexible sections that are open to interact with other PSD proteins. Interestingly, the N‐terminal segment, preceding the PH‐C2‐GAP region, emerges as a potential LLPS‐promoting region, although to our knowledge, only CC domain variants such as the L1202D and K1252D double variant have been suggested to affect LLPS so far [10, 11]. The complexity of the phenomenon is highlighted by the existence of alternative isoforms, where SynGAP isoform C is missing the entire N‐terminal region and even the PH domain is partially truncated [7]. Moreover, due to the inherent flexibility and mutational tolerance of IDRs, and the collective nature of LLPS, any single missense variant may not be sufficient to breach the functional threshold required for pathogenicity alone. This could be the case due to the presence of redundant or compensatory mechanisms, such as clusters of nearby phosphosites (Figure 5e and Tables S11–S12). Hence, the structured regions may indeed be more susceptible to pathogenic missense variation than the IDRs (Figure 4a vs. Figure 4b), and thus, the ClinVar‐submitted data and predictions could simply reflect this reality.

The PH‐C2‐GAP segment forms a tightly interconnected unit (Figure 1a,b) that typically remains relatively stable in nonbiased classical MD simulations, even with disease‐associated variants [46]. Regardless, the PH and GAP domains emerge as hotspots for pathogenic missense variation, showing high variant counts (Figure S5b), strong pathogenicity predictions and relative enrichment (Figure 4b and Tables S6 and S7) based on the AlphaMissense scoring. The C2 and CC domains also display notable pathogenic propensity. Variants altering CC‐mediated trimerization are likely pathogenic by affecting LLPS. Unlike the putative SH3‐binding motif, the NPxF motif that binds AIDA‐1 [9] appears highly sensitive to missense variation based on predictions (Figure 4b). Although recent findings suggest that the GAP domain may function largely as a structural element rather than as a canonical GTPase‐activating domain [81], the marked overlap between SRD variants and somatic cancer mutations remains noteworthy (*N* = 60). In particular, eight ClinVar‐listed and 18 cancer missense variants that map directly to the SynGAP‐Ras/Rap interface have been predicted in silico to potentially impair the complex formation and, thus, likely affect the GTP hydrolysis as well [82].

Moreover, modeling suggests that the C2 domain may contribute to SynGAP‐membrane association (Figure 1b), potentially aided by GAP‐GTPase interactions during localization [46]. This raises the possibility that mutations at the C2‐membrane‐water interface could be pathogenic (Figure 1c). Because simple structural distances have previously been linked to the effects of missense variants on protein interactions [83, 84], the potential of Euclidean distances from SynGAP residues to the Ras‐GTPase, bound GTP, and membrane as predictors of pathogenicity was evaluated. However, benign and pathogenic variants showed largely overlapping distance distributions, resulting in poor discrimination (Figure S8). Thus, these distance‐based metrics did not provide a useful structure‐based classification rule, although this issue should be reevaluated once more clinically assigned variants become available.

Finally, this study generated the SGM server (Figure 6a), providing comprehensive access to SynGAP missense variant data to support pathogenicity assessment. It integrates prior modeling content [46], including MD snapshots, in silico mutagenesis models, and extensive folding stability results. The server also provides a growing number of predictions for pathogenicity (*N* = 14; Table 1), phase separation (PSMutPred) [66], disorder/order (e.g., IUPred) [65] or disorder‐binding propensity (ANCHOR) [80], and links to ClinVar [3] and gnomAD [85]. Interactive tools such as the *3D Viewer* and *Data Table* (Figure 6b,e) make variant exploration intuitive, with structural views of SynGAP complexes with Ras‐GTPase and membrane (Figure 1d) toggleable in the viewer (Figure 6b,d). To enhance usability, AI‐based prediction summaries (Figure 6b) and 2D interaction plots for variants (Figure 6c) are provided as well.

## 4. Conclusions

Our conclusions were as follows:

1. A thorough comparison reveals that DL‐based methods (ESM1b and AlphaMissense) and folding stability calculations (e.g., Foldetta) provide the most accurate predictions for the *SYNGAP1*‐related disorders or SRD missense variant pathogenicity.
2. Due to its apparent and substantial untapped potential, AlphaMissense was given a *SYNGAP1*‐specific dual‐threshold optimization, which fully unlocked its capabilities, enabling AlphaMissense Optimized to outperform all other prediction methods at least in benchmarking.
3. Analysis of relative pathogenicity using AlphaMissense predictions highlights possible strong enrichment in specific domains: Most variants of the tiny NPxF motif are likely to be pathogenic, whereas the substantially larger GAP and PH domains also suggest high susceptibility to pathogenic missense variation.
4. Missense pathogenicity predictions exhibit a clear structural bias: variants predicted as pathogenic are concentrated in the ordered or domain regions, whereas benign predictions are more frequent in disordered or nondomain regions. This pattern is consistent across structure‐based and sequence‐based methods and is mirrored by clinically validated SRD‐ClinVar entries as well.
5. Missense variants predicted to alter phase separation and experimentally verified phosphosites appear to cocluster within the protein′s disordered regions, potentially defining phase separation‐promoting segments involved in LLPS at the PSD. The clustering extends beyond the C‐terminus and CC trimerization domain, raising the possibility of broader functional relevance across the protein that remains to be experimentally verified.
6. To support SRD missense variant interpretation, the SGM server offers a user‐friendly online platform for assessing the pathogenicity of *SYNGAP1* missense variants, integrating multiple predictive tools and structural insights for streamlined analysis.

## 5. Methods

### 5.1. SynGAP Missense Variants

The SGM server focuses on missense variants of SynGAP1‐*α*2 (isoform 1; UniProt: Q96PV0‐01). A comprehensive list of SynGAP missense variants was acquired from ClinVar (https://www.ncbi.nlm.nih.gov/clinvar/; *N* = 809; 05/25/2026; Figure 1d) [3], gnomAD v4 (Genome Aggregation Database; https://gnomad.broadinstitute.org/news/2023-11-gnomad-v4-0/; *N* = 1321; 05/25/2026) [86] and other resources (e.g., Mastermind (https://mastermind.genomenon.com/; 04/21/2023; *N* = 2; C233Y and E238K) [87]. With gnomAD, both the exome and genome sequencing–based variant data were included without filtering to acquire comprehensive list. All possible missense variants resulting from single‐base substitutions, including those not yet observed in public databases, were also included, bringing the final variant count to 8752.

### 5.2. Benchmark Test Set

For assessing the effectiveness of different pathogenicity prediction methods, ClinVar data was used to prepare a benchmark test set. The set was utilized to generate confusion matrices and calculate metrics such as precision, recall, and accuracy (Figure 2, Table 1, and Tables S1–S3; Eqs. 1–9 in the SI). The test set contained the benign (*N* = 75) and likely benign (*N* = 53) variants, as well as pathogenic (*N* = 21) and likely pathogenic (*N* = 42) variants from ClinVar [3], respectively. A few variants with conflicting assignments were categorized as benign (K492E, R575C, R775Q, T790N, E1286D, and T1305A) or pathogenic (R716Q and W572R) when their benign or pathogenic leaning submissions exceeded the VUS submissions (File S1). The remaining variants with conflicting (*N* = 89) and VUS status (*N* = 529) were combined under unclear category (*N* = 618). Note that not all pathogenicity prediction methods have by default an unclear (or comparable) category.

### 5.3. Molecular Modeling

SynGAP missense variants were generated via in silico mutagenesis using the human SynGAP‐AlphaFold model (AF‐Q96PV0‐F1‐model) [8] as previously described [46]. MODELLER10.3 [88] was used for variant modeling, followed by 150‐ns MD simulations with AMBER22 [89]. The inner leaflet membrane‐SynGAP model and SynGAP‐RasGTPase complex (Figure 1b) were built using CHARMM‐GUI [90] and the GAP‐Ras template (PDB: 1WQ1 [72]), and MD simulated with GROMACS2021 [91]. These interactions can be visualized in the *3D Viewer* via “*Add membrane*” or “*Add RasGTPase*” (Figure 6b,d). MD‐based annotations classified 211 variants as potentially pathogenic, benign, or uncertain based on visually observed structural impacts. Folding stability was assessed using FoldX‐MD [38], Rosetta [54, 59, 60, 92, 93], PremPS [57], and Foldetta [56], which integrates FoldX and Rosetta for consensus scoring (Figure 2). For further details, see the SI.

### 5.4. Server‐Based Predictions

Online server‐based predictions were done using several online tools, including SIFT [39], PROVEAN [49], PolyPhen‐2 [47], ESM1b [41], AlphaMissense [42], FATHMM [48], REVEL [40], EVE [51], and CADD [50] to predict the impact of missense variants for the *SYNGAP1* gene. The missense variant phase separation and disorder predictions were acquired via PSMutPred (http://psmutpred.online/)%5b80 [66] and IUPred2A (https://iupred2a.elte.hu/) [65], respectively. The phosphosite information was acquired from PhosphoSitePlus [67] (https://www.phosphosite.org/). The prediction tools rely on various methodologies such as ML, DL, Markovian modeling, and ensemble approaches to assess the pathogenicity of the missense variants. They can use structure and/or sequence information to classify variants as benign or pathogenic as well as in‐between/unclear. For example, SIFT assigns variants as affected or tolerated based on sequence homology, whereas PolyPhen‐2, which considers the impact based on protein sequence and structure, assigns variants as possibly damaging, probably damaging, or benign. For further details, see the SI.

### 5.5. Threshold Optimization

SynGAP‐specific threshold calibration or optimization was performed for all pathogenicity prediction methods using an exhaustive search over every unique candidate cutoff observed among clinically labeled test‐set variants with an available score. Single‐threshold models made direct benign or pathogenic calls, whereas dual‐threshold models used separate benign and pathogenic cutoffs and classified intermediate scores as uncertain. Thresholds were selected by maximizing Youden′s *J* on the test set, with dual‐threshold solutions required to classify at least 80% of clinically labeled test‐set variants with an available score. For the folding‐stability methods, the primary optimization analysis was restricted to a single destabilizing threshold, whereas the established multi‐interval thresholds were retained for the default, nonoptimized classifications. Because AlphaMissense performance improved substantially following dual‐threshold optimization (Figure 2 vs. Figure 3) and the optimized thresholds remained stable in the stratified five‐fold cross‐validation (Figure S3), the calibrated model was included in the SGM server as “AlphaMissense Optimized”, alongside the default “AlphaMissense” results. CADD thresholds were similarly calibrated and validated because the method requires gene‐specific calibration before usage. All optimized thresholds should be reevaluated as additional clinically validated variants become available.

### 5.6. AI‐Generated Summaries

AI‐generated summaries of pathogenicity predictions are provided for all missense variants included in the SGM server. These were generated using OpenAI gpt‐oss20B language model (License: Apache 2.0; https://openai.com/index/introducing-gpt-oss/) via Ollama0.11.4 (License: MIT; https://github.com/ollama/ollama). Gpt‐oss‐20b was retrieved with Ollama 08/21/2025. All prediction results, in simplified benign/pathogenic/uncertain format, were processed and incorporated into the ≤ 200‐word summaries to assess variant pathogenicity. In the prompt, emphasis was put on the significance of AlphaMissense‐Optimized, Foldetta, and general prediction consensus due to their high balanced accuracy (Table 1). The prediction summaries are accessible through both the *Data Table* and the *3D Viewer*, offering users a clear and interpretable overview of variant status.

### 5.7. Coding for the Server Webpage

The SGM server (https://syngapmissenseserver.utu.fi; Figure 6) was coded using HTML, CSS, PHP, JavaScript, and MariaDB. The *3D Viewer* (Figure 6b), presenting the SynGAP variant 3D structures, is based on MOL ^∗^4.10. 1 [70]. The *Data Table* (Figure 6e), which presents analysis metrics in tabular form from various structure‐ and sequence‐based methods, was set up as a database. All data, including variant structures, MD snapshots, data plots (Figure S6), and prediction data are downloadable. User privacy is respected as no user data, including IP addresses, is collected. The SGM server, tested using multiple web browsers (e.g., Firefox, Microsoft Edge, Google Chrome), is optimized for laptops or desktop computers. A three‐button mouse with scroll wheel in the middle is recommended for optimal user experience.

*3D Viewer functionalities* of the SGM server include

• *Navigation.* Users can select variants from a pull‐down menu or search box (e.g., L402R or c.1205T>G; Figure 6b). The MOL ^∗^ [70] *3D Canvas* view centers on the mutated residue, with options to refocus or navigate between simulation snapshots (< >; 0 ns, 50 ns, 100 ns, 150 ns Figure 6b) and replicas (1‐3, Figure 6b), if available. The rest of the variants with the structural template in the N‐terminal model are also available to provide structural perspective.
• *SynGAP-membrane association.* Visualize the inner postsynaptic membrane orientation relative to the N‐terminal SynGAP model using the *Add membrane* option (Figure 6b,d).
• *SynGAP-RasGTPase association.* Compare missense variant structures with Ras‐GTPase positioning using the *Add Ras-GTPase* option (Figure 6b,d).
• *Variant data*. With the MD simulated missense variants [46], a detailed written description of the structural effects of the missense variants is provided (Figure 6b). Variant‐specific pathogenicity predictions (Table 1 and Figure 1f) and analysis metrics are also included (Figure 6e), containing 2D images depicting the residue substitutions and variant interaction diagrams, AI‐generated prediction summaries, and MD‐related analysis metrics or data plots (Figure S6a–f). Downloadable 3D models are available.

*Search and support functionalities* of the SGM server include

• *Variant searches* can be performed via the landing page (Figure 6a), *Data Table* (Figure 6e), or *3D Viewer* (Figure 6b) using protein‐level changes (e.g., L402R; Figure 6b) or by nucleotide HGSVc codes (e.g., c.1205T>G). These codes correspond to the *SYNGAP1* gene MANE (Matched Annotation from the NCBI and EMBL‐EBI) transcript with the GenBank Accession Number NM_006772.3 or the matching Ensembl Transcript ID ENST00000646630. Links are provided to ClinVar and gnomAD, offering clinical status, review status (1–3‐star grading), submission numbers, ID, allele count, and frequency for entries. Additional related research is accessible via the DOI section (e.g. [15],).
• *Supporting sections* prodive access to legal statements, related publications, software licenses, FAQs, contact information, data downloads, and server announcements (Figure 6a). The *Metrics* section explains prediction methods and metrics, whereas the *Video* section includes online material related to the server and SRD, and a video of the SynGAP‐Ras complex interacting with the postsynaptic membrane in a simulation (Figure 6d).

### 5.8. Figure Preparation

Figures were generated using MOL ^∗^4.0.1 [70], BODIL [94], VMD1.9.4a12 [95], and PYMOL2.3 [96]. The 2D variant interaction diagrams were made using LigPlot+2.3.1 [71].

## Author Contributions

Conceptualization: P.A.P. and O.T.P.; methodology: A.E.A. and P.A.P.; formal analysis: A.E.A., H‐R.M.O., and P.A.P.; investigation: A.E.A., H‐R.M.O., and P.A.P.; resources: P.A.P. and O.T.P.; coding: J.V.L.; data curation: A.E.A and J.V.L.; writing—original draft preparation: A.E.A. and P.A.P.; writing—review and editing: A.E.A., P.A.P., H‐R.M.O., J.V.L., M.J.C., and O.T.P.; visualization: A.E.A., H‐R.M.O., J.V.L., and P.A.P.; supervision: P.A.P.; project administration: P.A.P.; funding acquisition and initial concept: P.A.P. and O.T.P.

## Funding

This study was supported by the CURE SYNGAP1, Leon and friends e.V, Research Council of Finland (348983, 337530, 357910), Novo Nordisk Foundation (0075825, 0068926), Finnish Cultural Foundation (00250204), and European Regional Development Fund (A92111).

## Disclosure

All authors have read and agreed to the published version of the manuscript. The authors take full responsibility for the accuracy and integrity of the content presented in this publication.

## Conflicts of Interest

The authors declare the following conflicts of interest: O.T.P. owns stocks in Aurlide Ltd. The remaining authors declare no conflicts of interest.

## Supporting information


**Supporting Information** Additional supporting information can be found online in the Supporting Information (SI) section. Supporting Information. The SI includes Supporting Methods section that provides additional methodological details on the modeling of SynGAP missense variants, MD‐based structural annotation, folding stability calculations, and in silico pathogenicity predictions. It also includes equations (Eqs. 1–9) for the applied pathogenicity prediction performance metrics. The supplementary figures and tables cover multiple protein disorder predictions (Figure S1), predictor benchmarking and threshold calibration (Figures S2–S4; Tables S1–S3), variant distributions across SynGAP domains (Figure S5) and sequence (Figure S7), MD simulation analyses (Figure S6), variant proximity to the membrane, Ras‐GTPase and bound GTP (Figure S8), prediction correlation calculations (Tables S4–S5), AlphaMissense prediction densities and score strengths for specific domains and datasets (Figure S5; Tables S6–S8) and analyses of predicted phase separation effects and experimentally reported phosphorylation sites or their estimated colocalization (Tables S9–S11).

## Data Availability

All data are deposited directly into the Downloads section of the SGM server (Figure 6a). This includes the MD trajectory snapshots (initial model, 0 ns, 50 ns, 100 ns, 150 ns) for WT‐SynGAP and 211 missense variant systems from three replicate simulations given in the PDB format. The PDB file compatibility was tested with numerous 3D viewing or modelling software (e.g., MOL ^∗^4.0.185, VMD1.9.4a12115, PYMOL2.3116, and BODIL114). Note that in the multimodel structures, the frames were superposed by aligning the protein backbone C*α* atoms using VERTAA in BODIL and the nonpolar hydrogens were removed using PYMOL. The in silico–generated variant 3D models (not MD‐simulated) were also given as PDB files. Both the MD simulation (GROMACS and AMBER) and MODELLER parameter files are included to make it possible to replicate the work, if necessary. All the relevant MD analysis metrics, annotation and descriptions, sequence‐ and structure‐based pathogenicity predictions, including folding stability calculations and DL‐based predictions are available both via the *Data Table* (Figure 6e) and *3D Viewer* (Figure 6b) but also downloadable as an all‐inclusive CVS table. Moreover, all the MD simulation analysis plots (e.g., RMSD; Figure S14a,b) can be downloaded via the server′s *3D Viewer* individually. The necessary data and information or instructions are provided online for replicating the SGM server build or generating another online server with matching functionalities and features. Zenodo (https://zenodo.org/) provides specific DOI links to the code in GitHub (https://github.com/) or ZIP packages for the SGM database schema (doi:10.5281/zenodo.17877274; License: ODbL‐1.0), MOL ^∗^ version 4.10.0 fork (doi:10.5281/zenodo.14653040; License: MIT), and main webpage (doi:10.5281/zenodo.17896548; License: GPL‐3.0). The server data, which is available online at the SGM server (https://syngapmissenseserver.utu.fi), was generated using various computational tools with different licenses, all of which allow at least their academic and noncommercial research usage. Therefore, the SGM server users are solely responsible for adhering to their specific license terms, especially in any potential commercial use cases. For further technical information, please contact the authors. Lastly, the Supporting Information contains additional figures and tables as well as relevant equations and Supporting Methods section with further details.
