# Supplementary material for: In Silico Assessment of SYNGAP1 Missense Variant Pathogenicity
Source: Hum Mutat. 2026 Nov 8;2026:8234670. doi: 10.1155/humu/8234670 (PMC13745803; doi:10.1155/humu/8234670)
Supplement: Supplementary file 1 [file HUMU-2026-8234670-s001.pdf]

Supporting Information for

In silico Assessment of SYNGAP1 Missense Variant Pathogenicity

Supporting Methods

Molecular modelling and in silico mutagenesis

The SynGAP missense variants were generated in 3D using in silico mutagenesis as described in a prior study[1]; and, thus, the steps are only briefly outlined here. The human SynGAP-AlphaFold model (AF-Q96PV0-F1-model_v3.pdb; https://alphafold.ebi.ac.uk/; 22/01/2022)[2]) was edited to include a continuous part with mostly ≥50 reliability (res. 198-730; Figure 1b). The variants in this 3D template were generated using MODELLER10.3[3] (Figure 1c). 211 variants were generated in a prior study[4], where they were probed for 150 ns in three replicate atomistic molecular dynamics (MD) simulations in solvent using AMBER22[5].

From these simulations, four production snapshots (0 ns, 50 ns, 100 ns, 150 ns) were extracted using CPPTRAJ in AMBERTOOLS22[6] and included as alternative models into the server (Figure 6b). Simulation trajectory analysis such as RMDS analysis (Figure S7a-b) or SASA were performed using CPPTRAJ. Ramachandran plots were generated using RAMAplot.py (GitHub: https://github.com/schlessinger-lab/RAMAplot; GNU Affero General Public License v3.0; accessed 01/11/2024) with MD snapshots (Figure S7e). The 2D protein secondary structure topology diagrams were plotted for 150 ns snapshots using SSDraw (GitHub: https://github.com/ethanchen1301/SSDraw)[7] (Figure S7f) and coloured using the normalized B-factor calculated over the entire simulation for each residue (Figure S7c-d).

The approximate position of the inner leaflet membrane relative to the SynGAP model (Figures 1b and 6d) was acquired by superposing the SynGAP-C2 domain using VERTAA in BODIL[8] against another C2-containing protein with established membrane association at the OPM[9,10]. The complex was generated using CHARMM-GUI[11] and simulated 300 ns using GROMACS2021[12] in a prior study[4]. Likewise, the approximate position of RasGTPase relative to SynGAP (Figures 1b and 6c) was acquired by superposing it against the human GAP-Ras complex structure (PDB: 1WQ1[13]) and its stability was probed in 130 ns MD simulation. Both the membrane and Ras positions can be visualized in the *3D Viewer* using "*Add membrane*" or "*Add RasGTPase*" options along with the missense variant 3D coordinates (Figure 6b,d).

Molecular dynamics-based annotation. The 211 SynGAP missense variants simulated in a prior study[1], were divided into potentially pathogenic and potentially benign groups based on their structural effects. Mutations with no discernible effects on the protein folding were deemed benign. Also, variants potentially affecting SynGAP-Ras or SynGAP-membrane complex formation were included in the uncertain group. The structural effects were itemized based on six criteria under the SynGAP Structural Annotation section in the *Data Table*:

1. *Secondary*: Missense mutations disrupting secondary structure elements (including β-hairpins, β sheets, α helices, and loops/turns) are potentially pathogenic.
2. *Tertiary Bonds*: Missense mutations altering tertiary structure bonding, particularly especially disrupting or forming hydrogen bonds or salt bridges between secondary structure elements, are potentially pathogenic.
3. *Inside-Out*: Missense mutations causing buried residues to become solvent-exposed and/or misplaced are potentially pathogenic.
4. *GAP-Ras Interface*: Missense mutations that may alter or disrupt the GAP domain and RasGTPase association are noted but not used as a criterion for pathogenicity.
5. *At Membrane*: Missense mutations that may alter or disrupt the C2 domain and membrane association are noted but without using it as criterion for assigning pathogenicity.
6. *No Effect*: Missense mutations unlikely to cause detrimental effects on protein structure based on simulations are potentially benign.

Protein folding stability calculations in detail

FoldX calculates the relative free energy of folding between the WT and missense variants[14]. The Gibbs free energy differences (ΔΔG values) are only comparable between the WT and variant system used in the calculation. This method, called the FoldX-MD approach, is based on 150 ns WT simulations, where each missense variant structure was generated for every representative nanosecond. Averages from three replica MD simulation trajectories were computed. The energy barrier for the destabilizing mutations was set to ≥2.0 kcal and ≤-2.0 kcal for the stabilizing mutations. Variants within the energy barrier range from -0.5 to 0.5 were considered neutral or likely benign. Note that 211 variants were already previously processed[1].

Rosetta allows backbone flexibility in protein folding stability calculations and can explore different conformational ensembles from a single starting structure[15,16]. The Rosetta Cartesian ΔΔG protocol[17–21] calculates the ΔΔG between the provided WT structure and the Rosetta-generated missense variant structures. This method involves relaxing the 0 ns snapshot of the WT-SynGAP structure using the full-atom FastRelax protocol[22] for 50 repeats each. The lowest energy relaxed structure is selected as the WT reference for ΔΔG calculations using ref2015 scoring function in the cartesian space[16,23]. The reported ΔΔG values are the lowest energy difference between the reference WT and the Rosetta-generated variant ΔG values from five prediction iterations for each run [24,25]. The ΔΔG values were converted to Kcal/mol using the scaling factor 2.94[15,26]. Calculations were performed using customizable PYTHON wrapper RosettaDDGPrediction available under GNU General Public License v3.0, (https://github.com/ELELAB/RosettaDDGPrediction)[15]. The ΔΔG cut-offs for the destabilizing mutations were set to ≥2.0 kcal and ≤-2.0 kcal for the stabilizing mutations, whereas variants with ΔΔG values within -0.5 to 0.5 were considered neutral or likely benign.

Foldetta protein folding stability results for the missense variants are the mean ΔΔG value of FoldX and Rosetta. The consensus score "Foldetta" correlates more accurately with the functional impacts from deep mutational scanning experiments than FoldX and Rosetta individually in distinguishing between pathogenic and benign variants[27]. The Gibbs free energy (ΔΔG) cut-offs are: ≥2.0 kcal/mol for destabilizing mutations, ≤-2.0 kcal/mol for stabilizing mutations, and -0.5 to 0.5 kcal/mol for neutral or likely benign variants.

PremPS (https://lilab.jysw.suda.edu.cn/research/PremPS/) is a computational method that evaluates the effect of missense mutation on protein stability[28]. PremPS utilizes evolutionary- and structure-based features calculated using PROVEAN[29] and FoldX[14], and is parameterized on a balanced dataset with an equal number of stabilizing and destabilizing mutations. ΔΔG values between WT and missense variants were calculated using the 0 ns frame of the WT-SynGAP simulation as the reference WT structure. No discernible difference in accuracy was found when using multiple MD simulation frames, so a single reference was used (data not shown). The ΔΔG threshold cut-offs were set to ≥1.0 kcal for destabilizing mutations and ≤-1.0 kcal for stabilizing ones. Variants with ΔΔG values between -0.5 and 0.5 were considered neutral or likely benign.

Server-based in silico pathogenicity prediction methods in detail

SIFT (Sorting Intolerant From Tolerant) predicts whether an amino acid substitution affects protein function based on sequence homology and the physical properties of amino acids[30]. The default settings of SIFT Sequence tool were applied for human *SYNGAP1* isoform 1 sequence (UniProt: Q96PV0-1) predictions. The median conservation of sequences was three and the alignment file is provided in the server's Downloads section. Alternative alignments of closely related animal sequences and mammal sequences were processed; however, the default setup produced better accuracy based on ClinVar[31] data comparison (data not shown). A substitution is predicted to be damaging if the SIFT score (0 to 1) is ≤0.05 and tolerated if >0.05.

PROVEAN (Protein Variation Effect Analyzer v1.1) predicts the effect of protein sequence variations through a delta alignment score based on comparisons between the reference WT and variant versions of a protein query sequence[29]. The tool employs BLAST to collect the sequences for comparison from the NCBI NR protein database[32]. PROVEAN applies CD-HIT to cluster BLAST hits using a parameter set at 75% global sequence identity. Predictions are generated using a sequence set composed of the top 30 clusters of closely related sequences. The default score cut-off threshold of -2.5 is applied for binary classification of variants. Variants with PROVEAN scores below this threshold are classified as deleterious or pathogenic, while those with scores above the threshold are considered neutral or benign[29].

PolyPhen-2 (Polymorphism Phenotyping v2) predicts the possible impact of amino acid substitutions on the structure and function of protein based of physical and comparative consideration[33]. The predictions were made using both HumDiv and HumVar options. HumDiv is compiled from differences between all damaging alleles causing Mendelian diseases, and the non-damaging ones between human and mammalian orthologues. It recognizes mutations with drastic effects

from normal human variation useful for the diagnosis of Mendelian diseases. HumVar is trained on differences between human disease-causing mutations in UniProtKB and common human nsSNPs (MAF>1%) with no disease association, identifying mildly deleterious alleles as damaging. The pph-2 probability score ranges from 0 (benign) to 1 (damaging), with scores closer to 0 and 1 indicating high confidence in benign and probably damaging predictions, respectively. "Possibly damaging" indicates low confidence in deleterious predictions.

ESM1b (Evolutionary Scale Modelling) is a protein language model that leverages deep learning to predict the effects of missense variants on alternative protein isoforms[34]. By analysing evolutionary information encoded within amino acid sequences, ESM-1b provides insights into the potential pathogenicity of genetic variants. Variants with log-likelihood ratio (LLR) scores ≥ -7 are predicted to be “likely benign,” those ≤ -8 are predicted to be “likely pathogenic,” and scores between -7 and -8 are “in-between”.

AlphaMissense predicts the effects of missense mutations on protein structure and function[35]. The DL model uses the AlphaFold2 model from Google’s DeepMind[2] as a base and incorporating information from multiple sequence alignments and genetic databases. Missense variants scoring 0 to 0.34 are likely benign, scores between 0.34 and 0.564 are ambiguous, and scores above 0.564 are likely pathogenic.

FATHMM (Functional Analysis Through Hidden Markov Models) is a species-independent method with optional species-specific weightings for predicting the functional effects of protein missense variants[36]. Coding variants were analysed using the disease-specific option: Nervous System. Missense variants that score >2.5 are benign, while those scoring ≤2.5 are pathogenic.

CADD (Combined Annotation Dependent Depletion)[37] is a machine learning method integrating multiple genomic annotations to predict the deleteriousness of coding and non-coding genetic variants. CADD scores are reported on a PHRED-scaled scale, where higher scores indicate greater predicted functional impact and pathogenic potential.

*EVE (*Evolutionary Model of Variant Effect; downloaded 25/06/2026)[38] is an unsupervised deep learning model that quantifies the evolutionary constraint on protein sequences from multiple species. By modelling natural sequence variation, EVE evaluates the potential impact of missense variants and generates scores from 0 (most benign) to 1 (most pathogenic).

REVEL (Rare Exome Variant Ensemble Learner) is an ensemble-based method for assessing the pathogenicity of missense variants[39]. It combines scores from 13 prediction tools including FATHMM v2.3[36], PolyPhen-2[60], SIFT[30], and PROVEAN[29]. The score ranges from 0 to 1 and with variants scoring ≥0.5 assigned as pathogenic. The pre-calculated predictions (v1.3 05/03/2021) in REVEL are not explicitly for the SynGAP1-α2, but of other transcripts of the *SYNGAP1* gene. Predictions for missense mutations at the equivalent GRCh38 positions are included in the *Data Table*.

Equations for assessing prediction accuracy and relatedness

Precision is a metric that tells how often the positive predictions are correct. It is calculated by dividing the number of correct predictions (true positive; TP) by the number of positive predictions (TP + FP) using the following equation (Eq.):

$$Precision = \frac{TP}{TP + FP} \tag{1}$$

Recall (sensitivity) is a metric that tells how often the method identifies positive instances correctly (TP) from all the actual positive cases. It is calculated by dividing the number of true positives by the number of positive instances a.k.a. including both true positive and false negative (TP + FN) using the following Eq.:

$$Recall = \frac{TP}{TP + FN} \tag{2}$$

Specificity is a metric that tells how often the method identifies negative instances correctly (TN) from all the actual negative cases. It is calculated by dividing the number of true negatives by the number of negative instances a.k.a. including both true negatives and false positives (TN + FP) using the following Eq.:

$$Specificity = \frac{TN}{TN + FP} \tag{3}$$

Accuracy is a metric that tells how often correct predictions, both positive and negative predictions, are made. It is calculated by dividing the number of correct predictions (TP + TN) by the number of all predictions (TP + FP + FN + TN) using the following Eq.:

$$Accuracy = \frac{TP + TN}{TP + FP + FN + TN} \tag{4}$$

Balanced accuracy measures the average accuracy of both positive and negative predictions by calculating the average of recall and specificity. It is calculated by calculating the mean value of recall and specificity using the following Eq.:

$$Balanced\ Accuracy = \frac{Recall + Specificity}{2} \tag{5}$$

F1 score is the harmonic mean of precision and recall that aims to balance precision-recall trade-off. It is calculated by calculating the harmonic mean value of precision and recall using the following Eq.:

$$F1\ Score = 2 * \frac{Recall * Precision}{Recall + Precision} \tag{6}$$

Matthews Correlation Coefficient (MCC) is a statistical tool for a comprehensive and balanced evaluation of binary systems. The prediction takes into consideration all the four confusion matrix sets (TP, FN, TN, FP). A perfect prediction gets a value of +1; a prediction made by chance receives a value close to 0; while -1 corresponds to a perfectly opposite prediction. The MCC was calculated using the following Eq.:

$$MCC = \frac{TPxTN - FPxFN}{\sqrt{(TP + FP)(TP + FN)(TN + FP)(TN + FN)}} \tag{7}$$

Phi (ϕ) coefficient is calculated using the following Eq.:

$$\Phi = \frac{bc - ad}{\sqrt{(a + b)(a + c)(b + d)(c + d)}} \tag{8}$$

where the possible prediction combinations included: *a* = benign, benign; *b* = benign, pathogenic; *c* = pathogenic, benign; and *d* = pathogenic, pathogenic. Note that MCC and ϕ coefficient are essentially the same, but, for clarity, these different terms are used.

R-squared (R^2) is calculated using the following Eq.:

$$R^2 = 1 - \frac{SSR}{TSS} \tag{9}$$

where SSR (Sum of Squared Residuals) stands for the sum of the squared differences between the observed and predicted values, whereas TSS (Total Sum of Squares) stands for the sum of the squared differences between the actual response variable values and the average response.

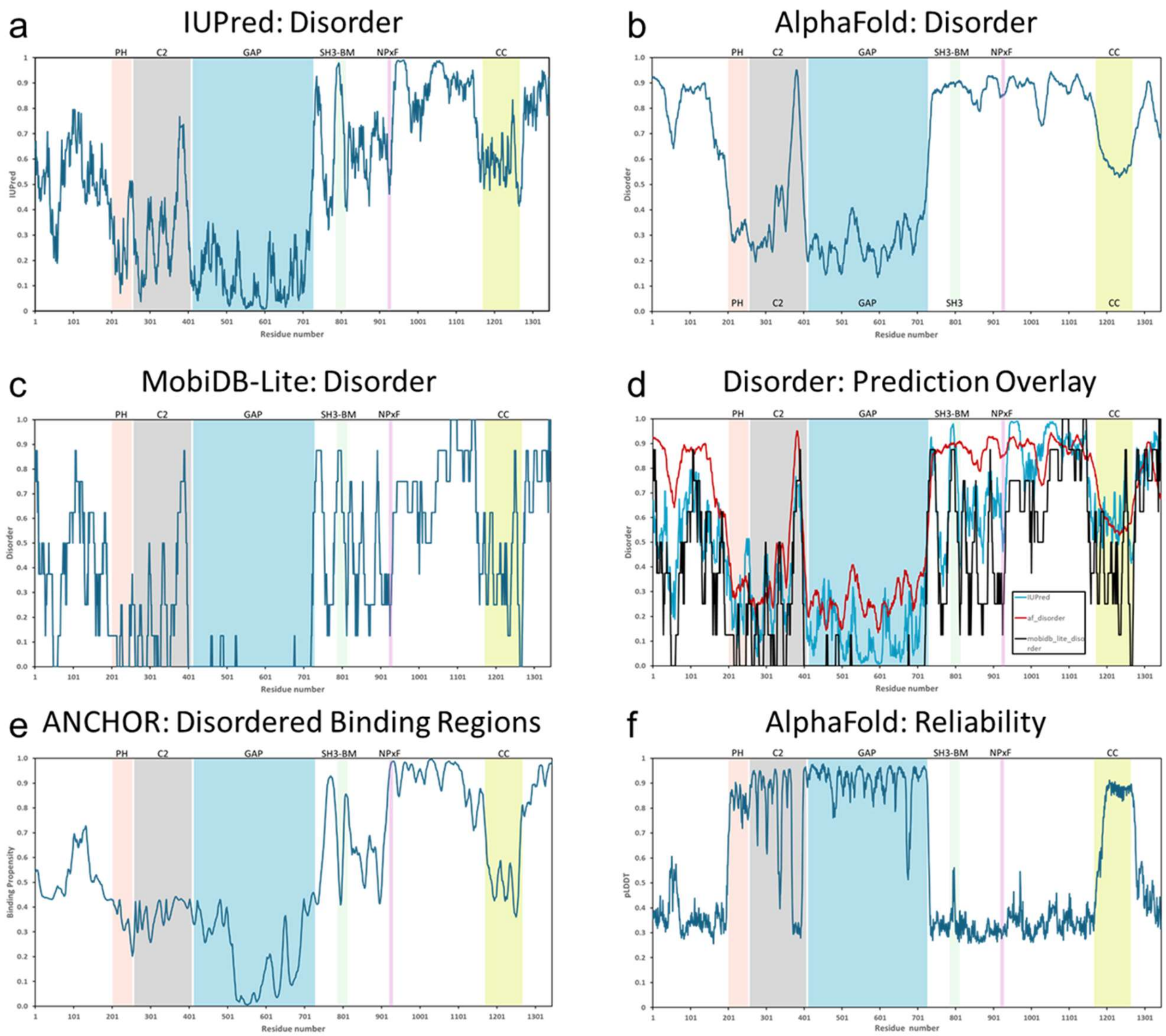

Figure S1. Disorder predictions for SynGAP. a) IUPred[40], b) AlphaFold disorder[35], and c) MobiDB-Lite[41], which is a consensus method for predicting long intrinsically disordered regions, provide consistent segmentation of SynGAP-α2 sequence into ordered and disordered regions. d) This agreement is emphasized by the overlay of disorder/order predictions, which closely mirrors both the domain architecture (coloured in the background) The predictions were downloaded from IUPred2A (https://iupred2a.elte.hu/; 09/30/2025)[41]. e) ANCHOR[42] produces a broadly similar curve to the disorder prediction methods (e vs. d); however, it focuses on identifying potential disordered binding regions such as short linear motifs or SLIMs rather than disorder per se. f) AlphaFold confidence scores (plod; Predicted Local Distance Difference Test) present an essentially opposite view to disorder predictions, in other words, regions with reliably predicted structure receive higher scores. Known domains and motif regions are indicated in the background. Known domains and motif regions are coloured in the background.

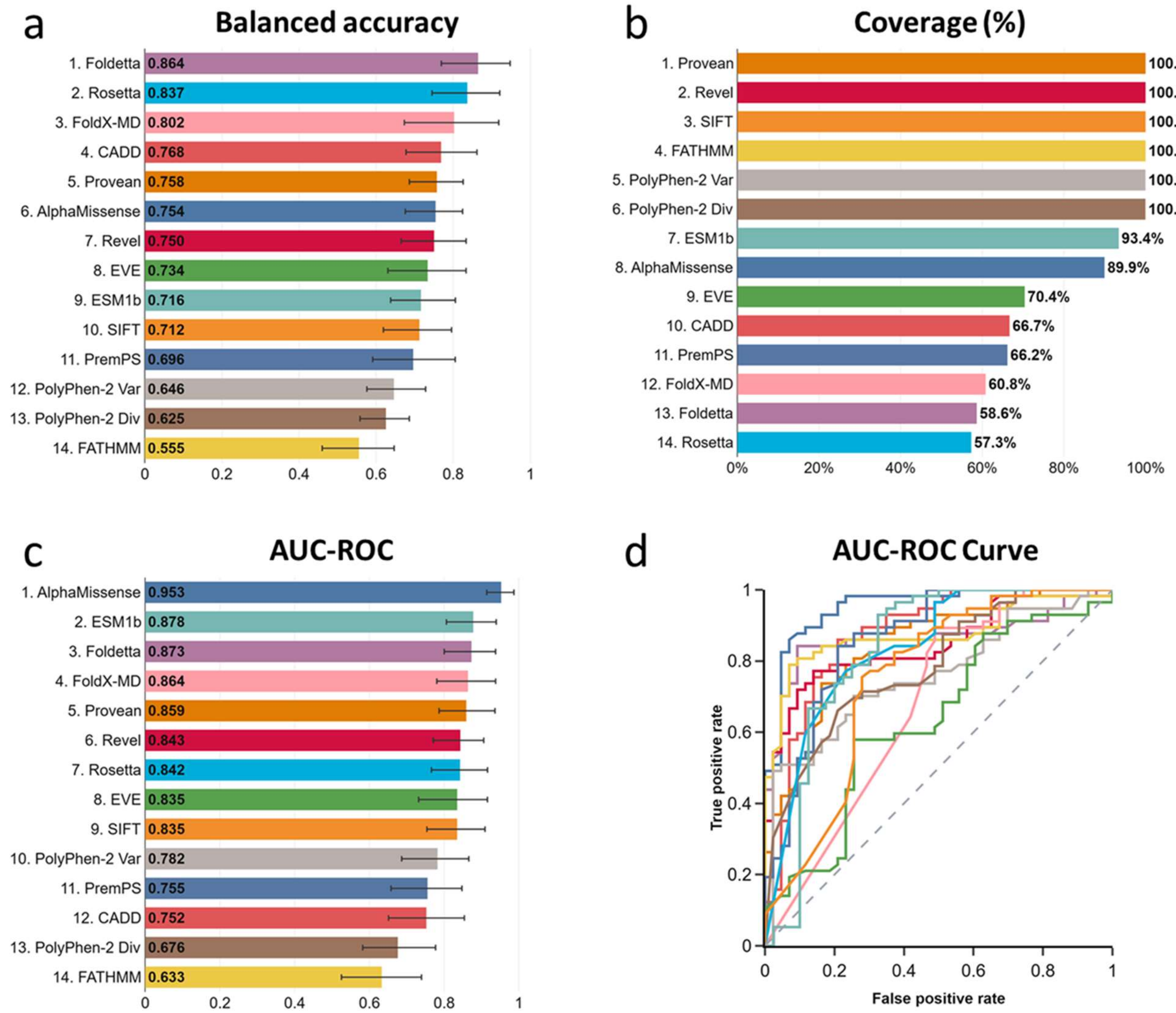

Figure S2. Benchmarking in silico predictors using clinical *SYNGAP1* variant data on the N-terminal PH/C2/GAP structure region. a) Balanced accuracy values of prediction methods are shown for the N-terminal structure region missense variants. b) Coverage % values of prediction methods are shown for the N-terminal structure region missense variants c) The AUC values and (d) the ROC curves indicate that when focusing on the structural range of the N-terminal SynGAP model (Figure 1b-c; res. 198-730) AlphaMissense[35] is better at recognizing pathogenic variants as TP than the other methods. This includes the FoldX-MD approach that has solely structural focus in the categorization. The folding stability results are shown for FoldX-MD, Rosetta, and Foldetta for comparison. All performance metrics are given as bootstrap means (from 5,000 iterations) and confidence intervals. For further information see Figure 2.

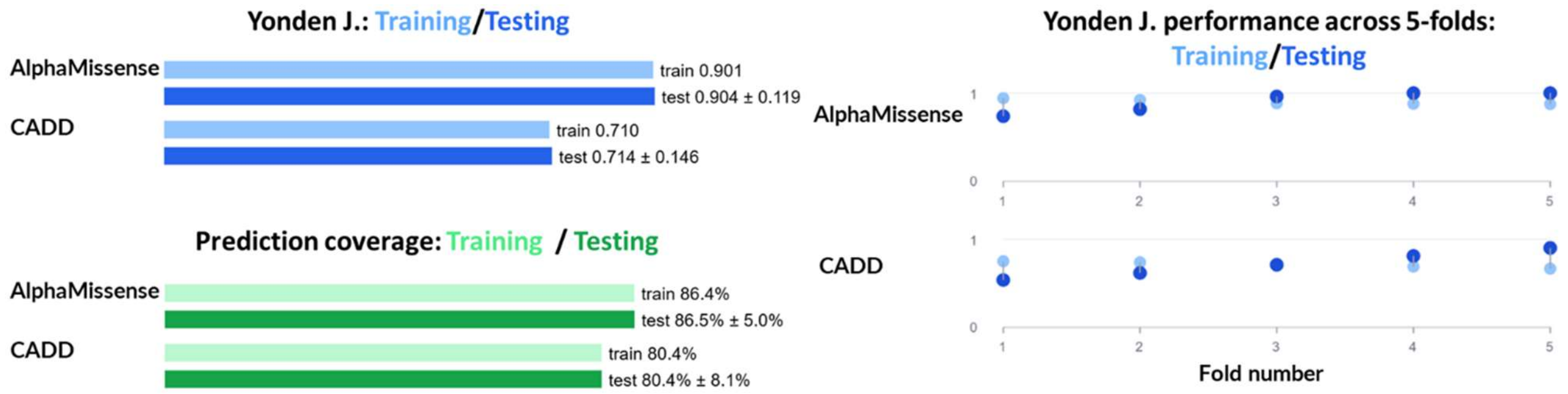

Figure S3. Five-fold cross-validation of *SYNGAP1*-calibrated AlphaMissense and CADD dual thresholds. The left side compares training and testing Youden J. values and prediction coverage, with testing values reported as mean ± standard deviation across folds. AlphaMissense showed high and closely matched training and testing performance (0.901 and 0.904 ± 0.119, respectively), whereas CADD showed lower but similarly consistent performance (0.710 and 0.714 ± 0.146). Prediction coverage also remained similar between training and testing for both methods. The right side shows training and testing Youden J. values for each fold, illustrating the consistency of threshold performance across the five cross-validation splits.

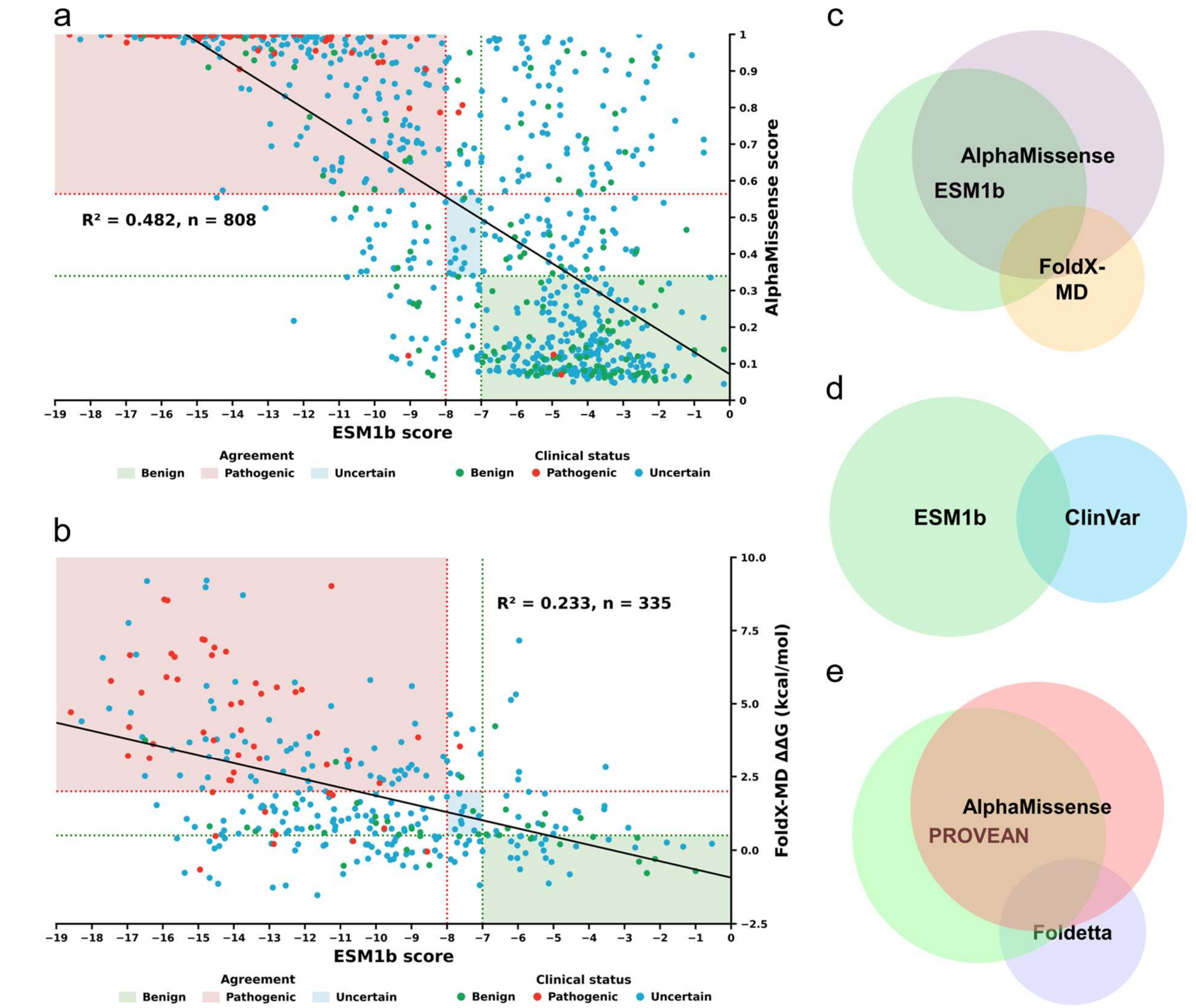

Figure S4. Estimating the pathogenicity prediction overlap of missense variants between different methods. a) When AlphaMissense[35] and ESM1b[34] scores are plotted against each other, the prediction methods can mostly recognize correctly those missense variants assigned pathogenic status in the ClinVar database[31] (red dots; transparent red area); however, the benign variants (green dots; transparent green area) are more scattered. The R^2 value 0.47 (Table S2) indicates that the predicted results are different overall and adjusting the benign and pathogenic threshold limits, which are shown with green and red dotted lines, respectively, would not change the situation. b) Despite the low R^2 value of 0.23 (Table S3), the comparison of ESM1b to FoldX-MD[43,44] on the predictions focusing on protein's N-terminal structural region (Figure 1b-c; res. 198-730), both methods were able to recognize the pathogenic variants effectively (transparent red area), while there was less agreement on the benign variants. ESM1b generated nearly perfect separation for the pathogenic variants with the default threshold limits. The two outlier values for the variant W1315R (0.21, 0.66) and S668F (-15.05, 16.72) are not shown in panels a and b, respectively. c) The Venn diagram, drawn using DeepVenn (https://www.deepvenn.com/; accessed 06/12/2024)[45], shows that despite substantial overlap, the top three methods produced plenty of different predictions for the missense variants. For example, FoldX-MD calculations, working only on variants with counterparts in the N-terminal model structure (Figure 1b), produce far less predictions than AlphaMissense or ESM1b. d) The Venn diagram highlights the disparity between the amount of ESM1b predictions and missense variants currently listed in the ClinVar database. e) The Venn diagram shows that PROVEAN and AlphaMissense acquire substantial overlap; however, Foldetta is providing less similar predictions.

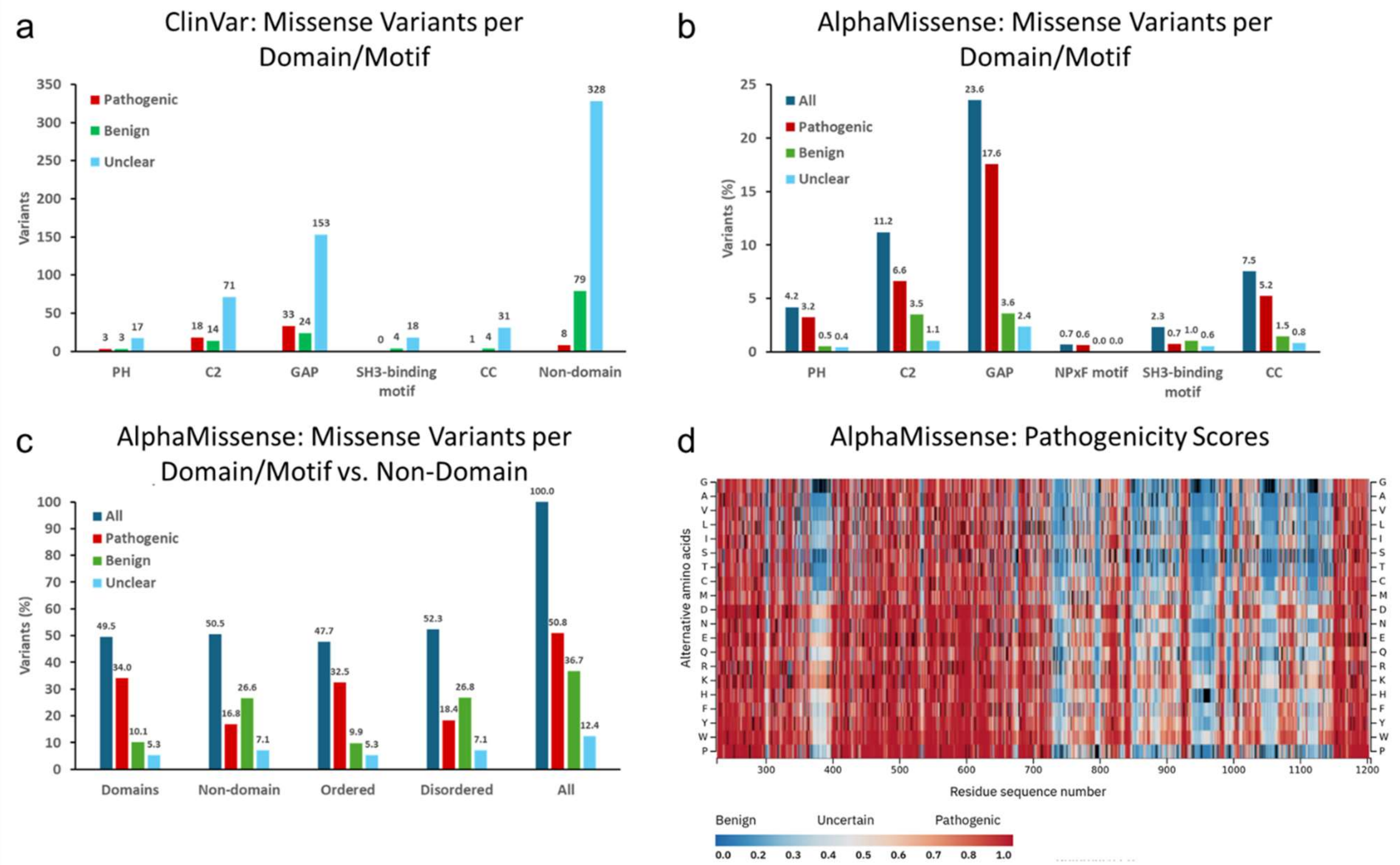

Figure S5. Domain-level distribution of SynGAP missense variants. a) Mapping of ClinVar-classified SynGAP missense variants onto annotated domains and motifs, grouped into simplified categories: pathogenic, benign, and uncertain significance. b) Domain distribution of all possible missense variants due to single-base substitutions, including those from ClinVar and gnomAD, predicted by AlphaMissense (applying default thresholds). c) Classification of AlphaMissense-predicted variants by domain vs. non-domain regions, and by structural context (ordered vs. disordered) as defined by IUPred. d) A heatmap of AlphaMissense scores for all possible missense variants retrieved from the AlphaFold Protein Structure Database (https://alphafold.ebi.ac.uk/; 10/29/2025) reveals elevated pathogenicity predictions in structurally reliable regions (see Figure 4a and 4b).

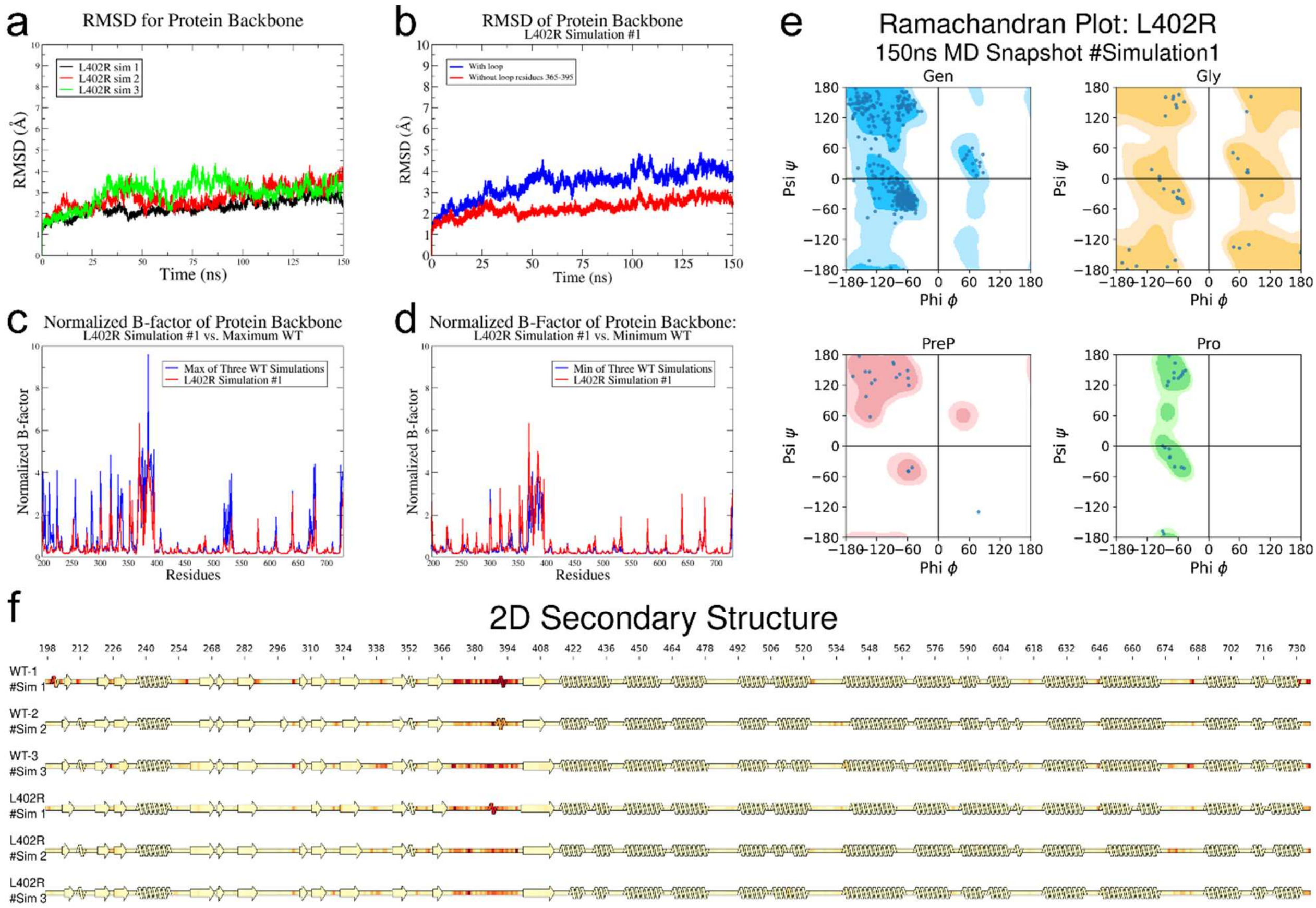

Figure S6. Molecular dynamics simulation analysis plots for missense variant L402R. a) RMSD (Root Mean Square Deviation) of the protein backbone is shown for the three 150-ns replica MD simulations of the L402R missense variant probed in solvent. The first simulation frame was used as a reference for the calculations with CPPTRAJ in AMBERTOOLS22[6]. The Ω Gly-rich loop was omitted from the calculations because of its excessive and arbitrary movement obscuring the results. b) The RMSD of protein backbone for one of the replica 150-ns simulation with the Ω Gly-rich loop included (blue) and excluded (red). c) The normalized B-factors of protein backbone[46] were calculated for all saved frames in the variant simulation (red) and plotted against the equivalent maximum values of three WT replica simulations (blue) for comparison. d) Likewise, the normalized B-factors of the variant simulations (red) were plotted against the equivalent minimum values of the three WT-SynGAP replica simulations (blue) for comparison. e) Four different Ramachandran plots were generated for the L402R variant simulation at 150 ns using RAMAplot.py (GitHub: https://github.com/schlessinger-lab/RAMAplot; GNU Affero General Public License v3.0; accessed 01/11/2024) and CPPTRAJ in AMBERTOOLS22[6]. The plots include specific ones for proline (Pro), one residue before proline (PreP), glycine residues (Gly), and all other residues (Gen). The plots were also included for 0 ns, 50 ns, and 100 ns frames but only the last frame is shown here for brevity. f) The 2D protein secondary structure topology diagram with B-factor colouring for the 150 ns snapshot depicts the locations of α helices (helical spring), β strands (arrows) and coils/loops/turns (lines). The topologies were plotted using SSDraw (GitHub: https://github.com/ethanchen1301/SSDraw)[7] and are coloured with Lord colormap in MATPLOTLIB using the side chain movement normalized B-factors calculated based on the entire 150-ns MD simulation.

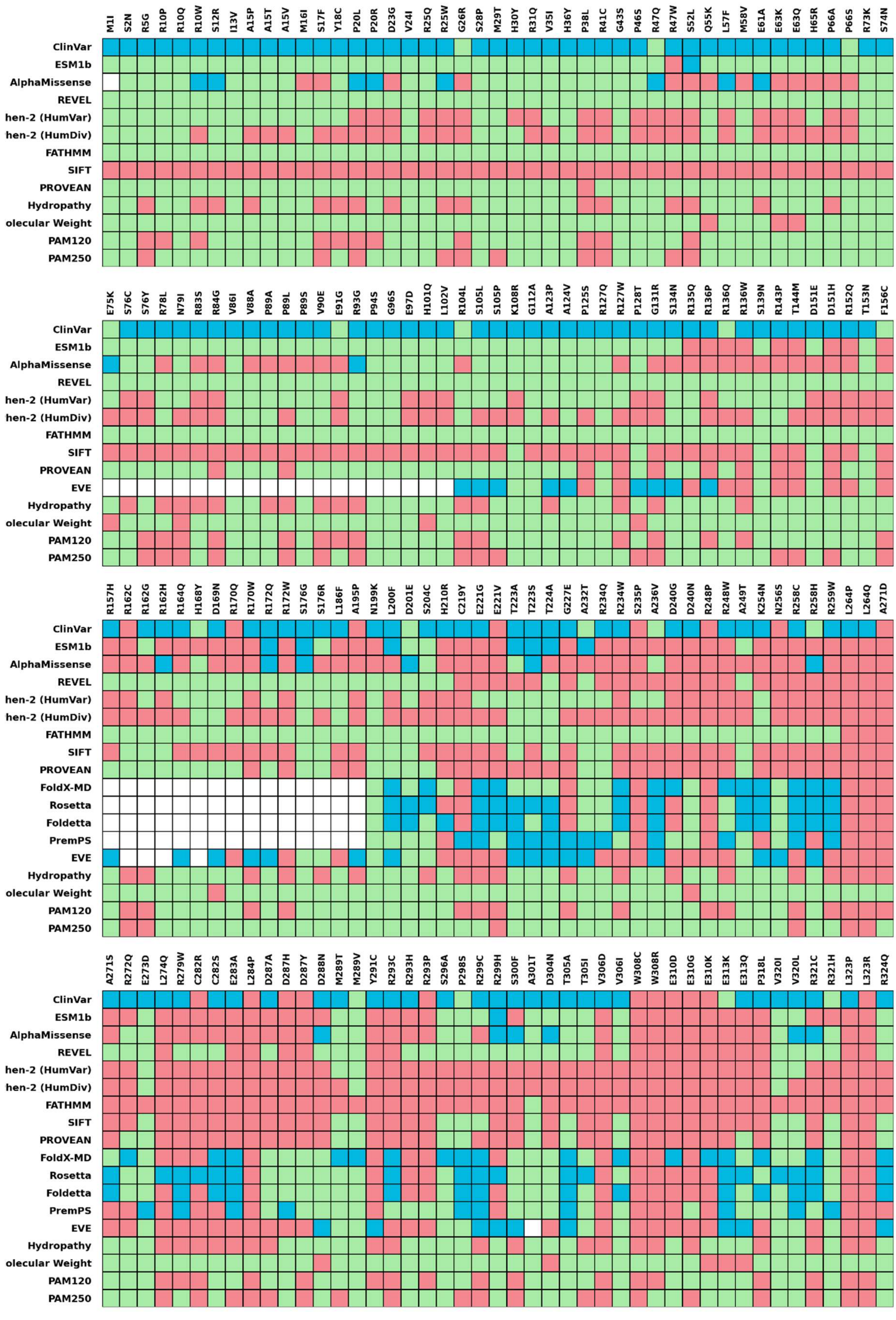

Figure S7. **Colour map for the SynGAP missense prediction methods and ClinVar.** The pathogenic (red), benign (green) and unclear (blue) SynGAP missense variants are coloured and those variants without prediction are left white.

Figure S7. Continuing.

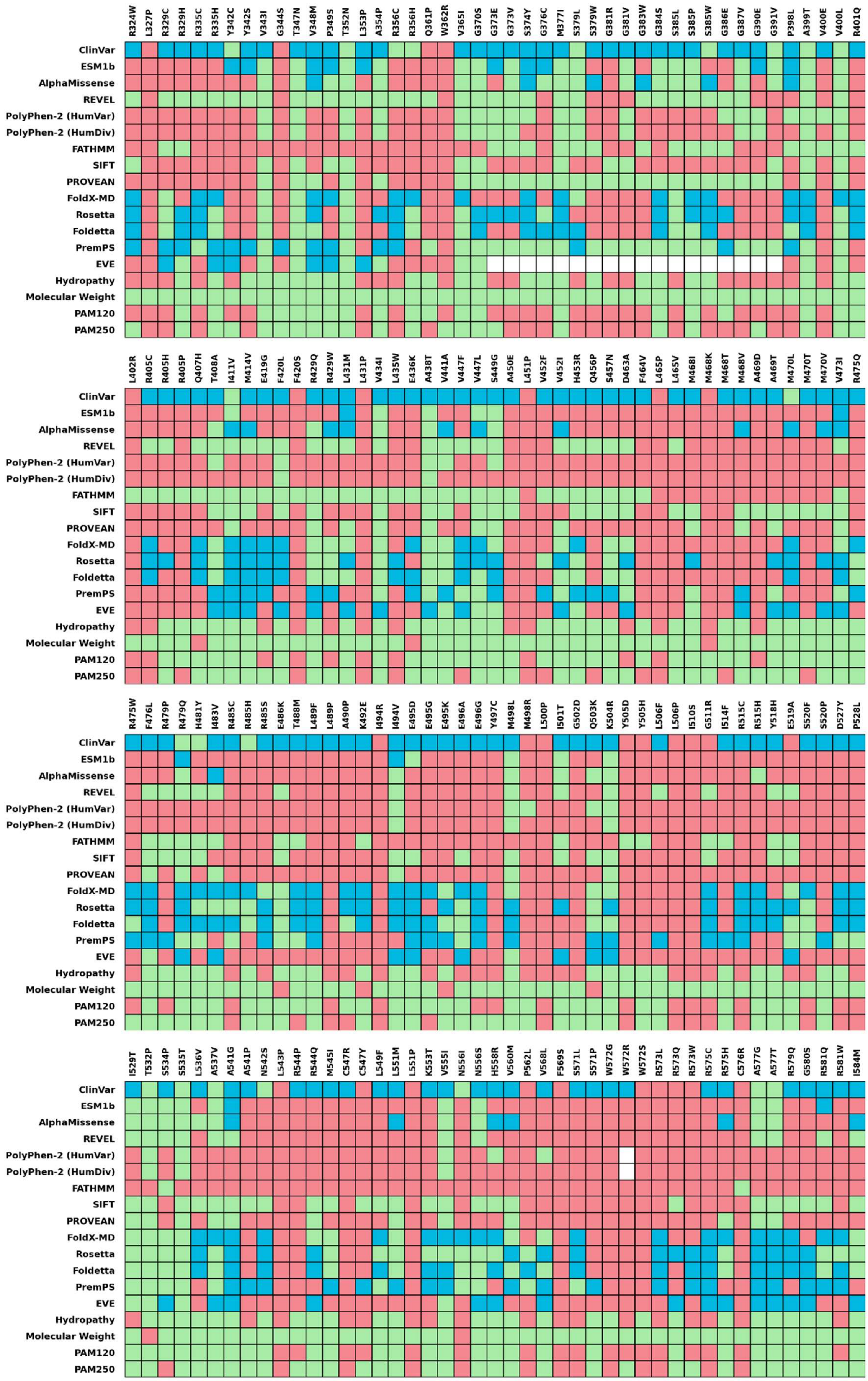

Figure S7. Continuing.

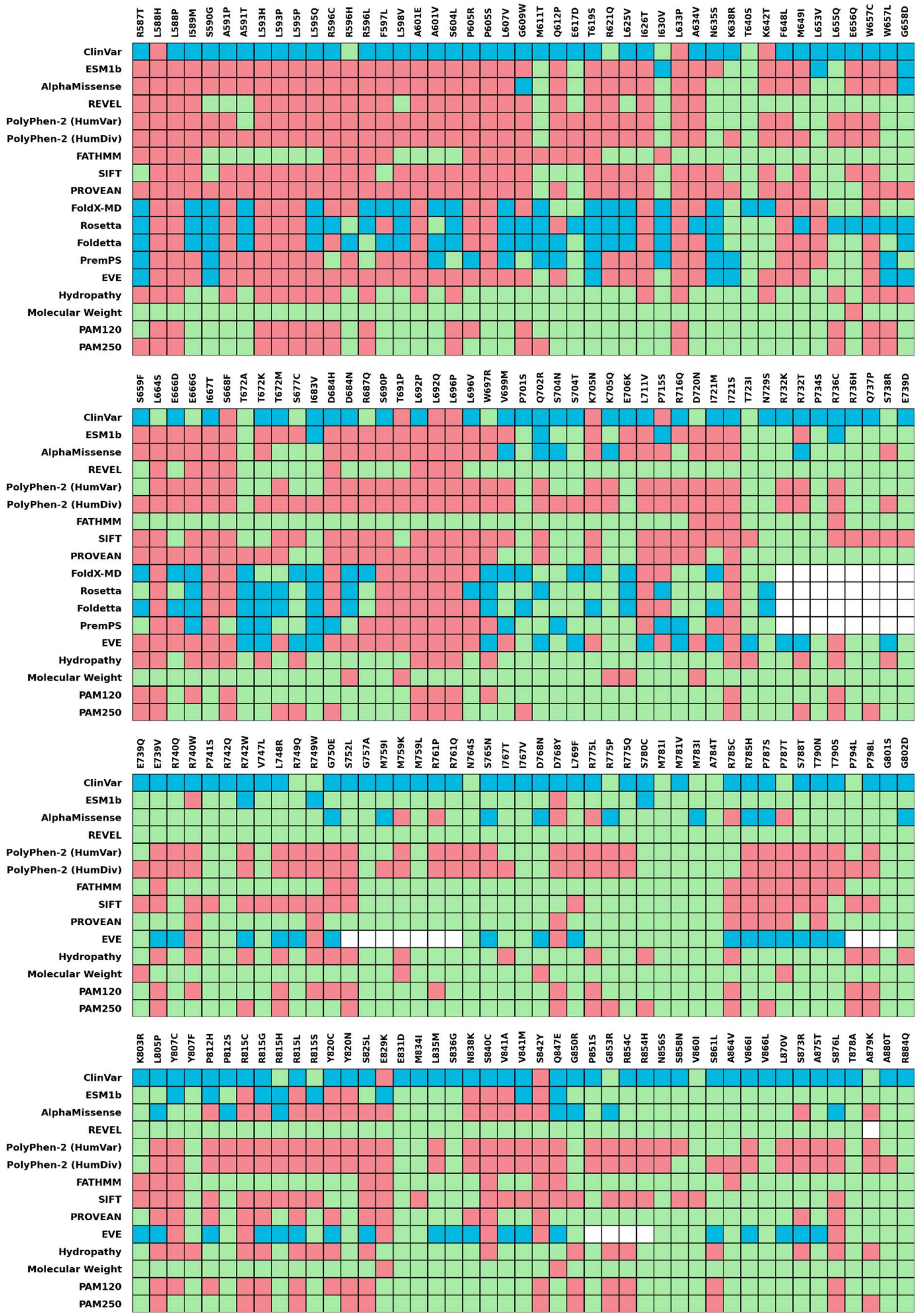

Figure S7. Continuing.

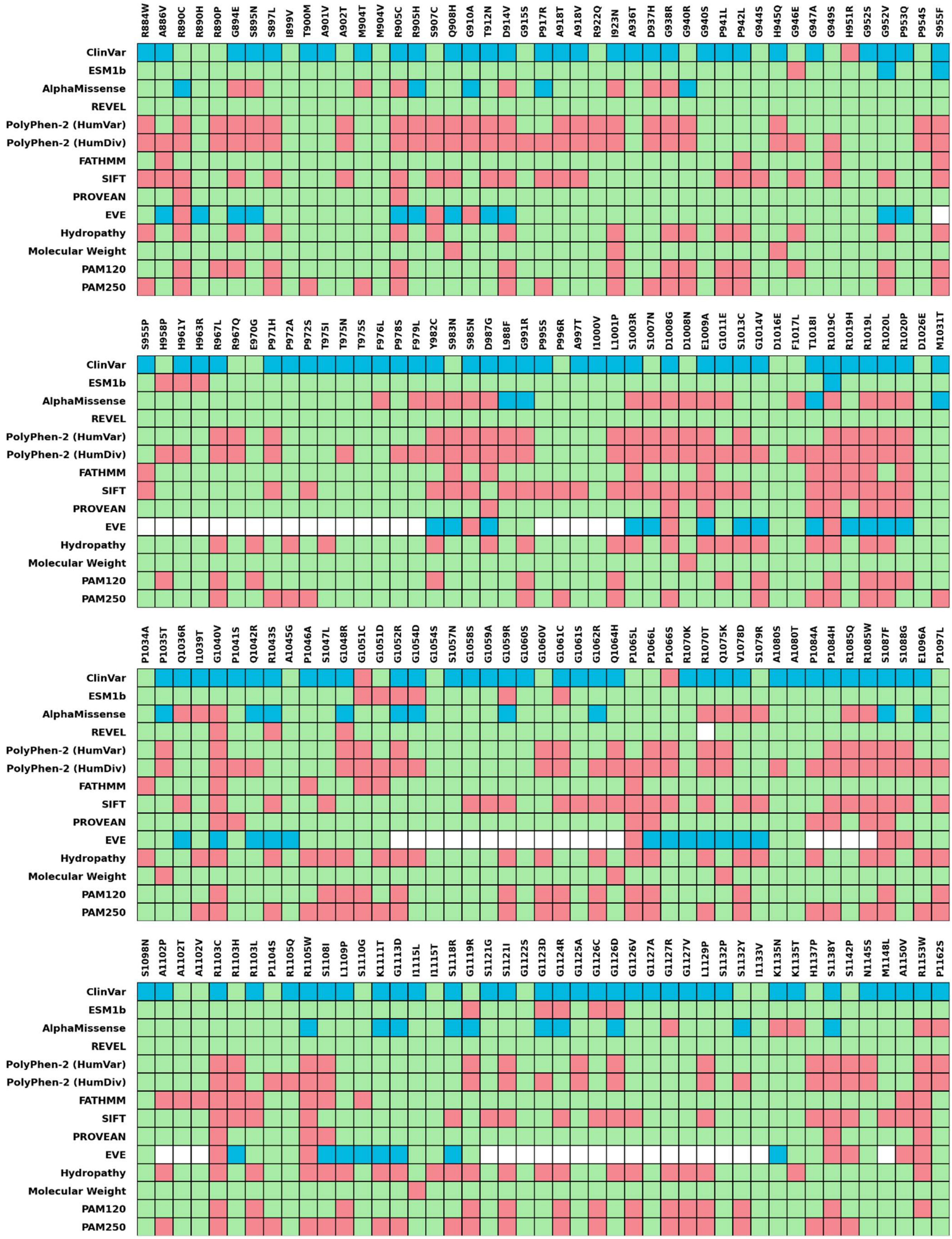

Figure S7. Continuing.

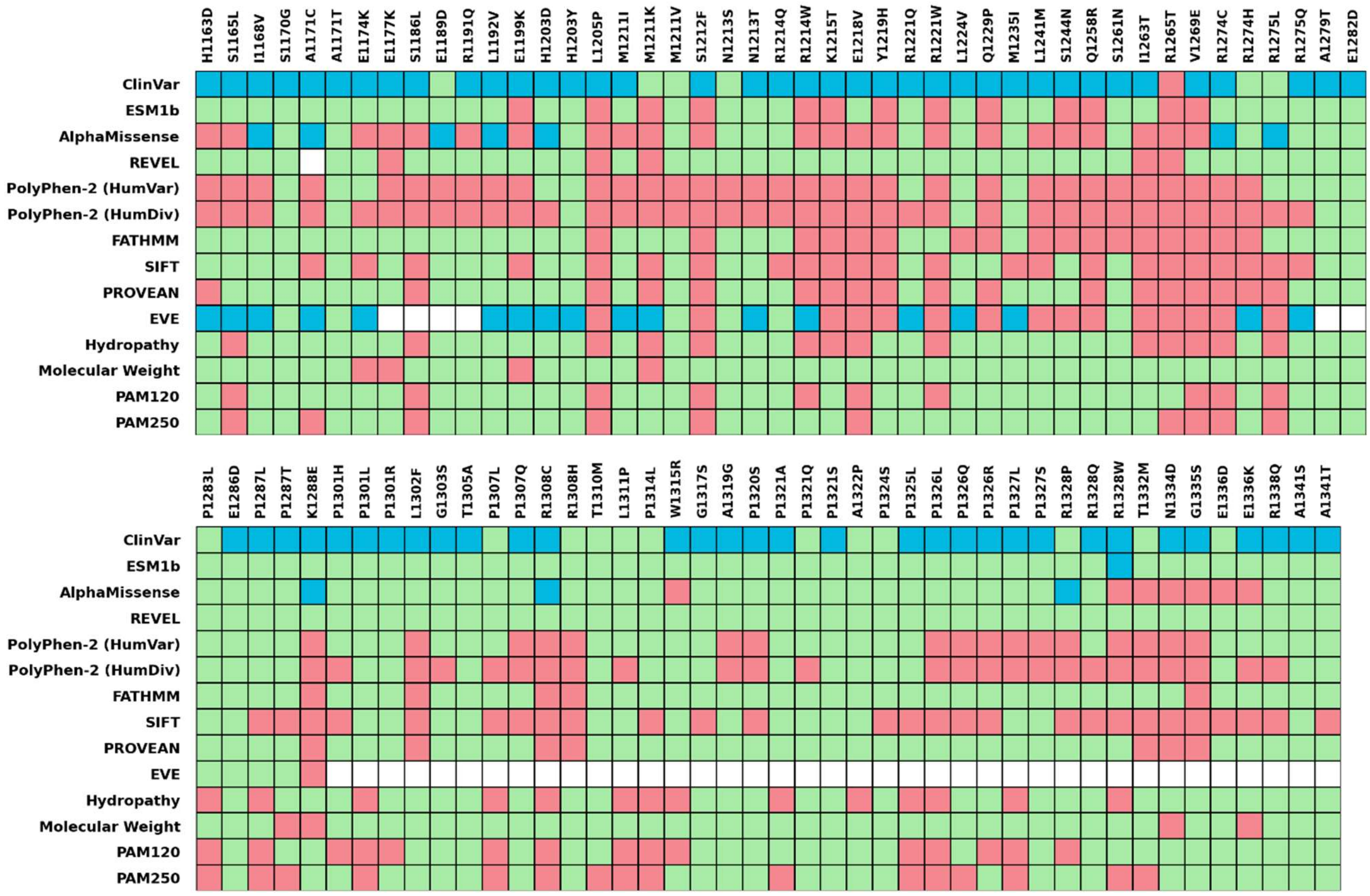

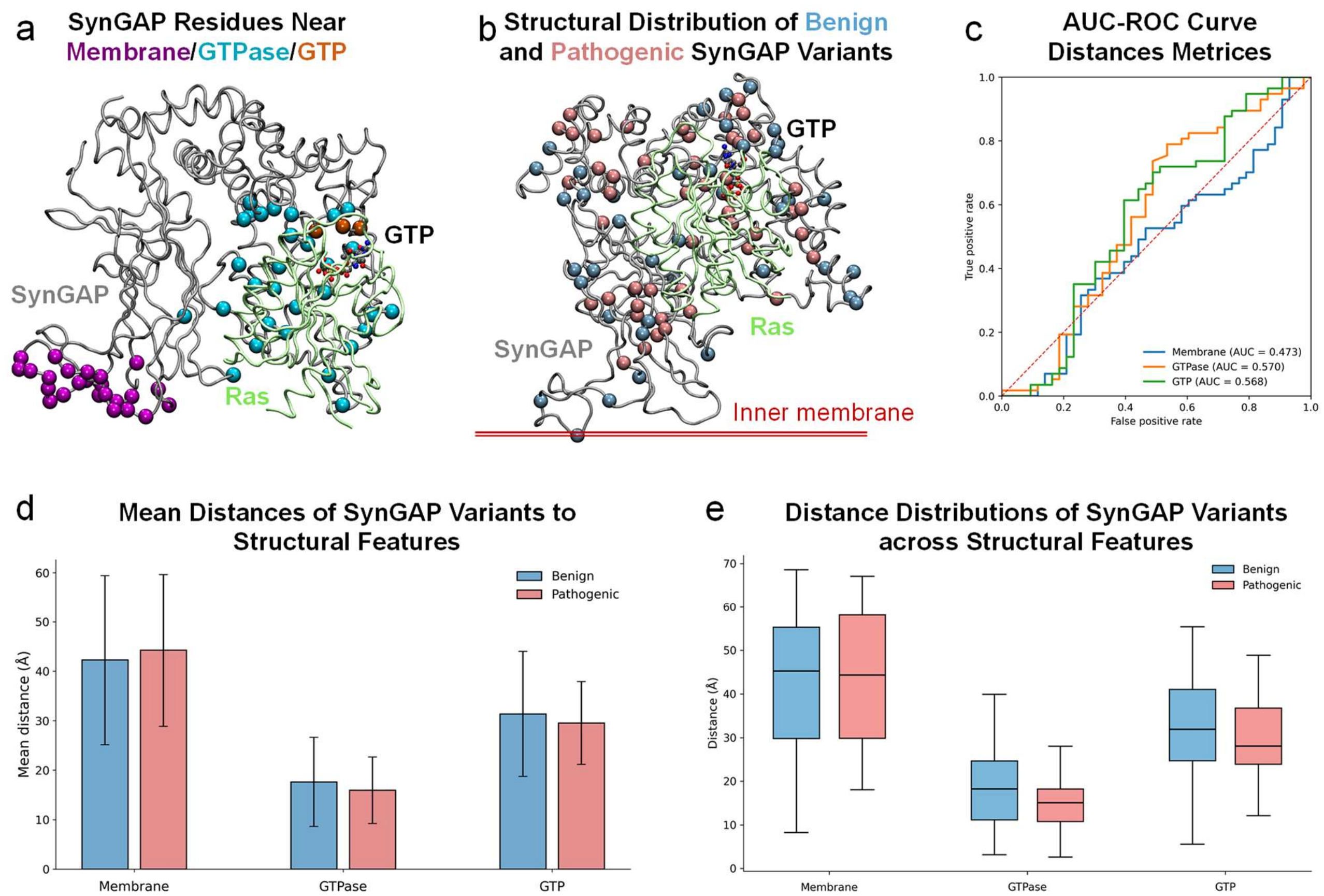

Figure S8. Distance-based mapping of SynGAP variant positions relative to the membrane, Ras, and GTP. a) Maps SynGAP residues that contact the membrane (magenta), the Ras GTPase (cyan), or both the GTPase and GTP (orange). SynGAP is shown as a silver tube, Ras as a green tube, and GTP in black CPK representation. Residues were classified as contacting when the minimum heavy-atom distance was ≤4 Å in at least 20% of analyzed frames. b) Shows the positions of benign (light blue) and pathogenic (light pink) SynGAP variants on the SynGAP–Ras complex. The approximate inner membrane position is indicated by the red line. c) Evaluates discrimination using ROC curves for distance to the membrane (blue, AUC = 0.473), GTPase (orange, AUC = 0.570), and GTP (green, AUC = 0.568). These distance metrics showed no or only very weak discrimination between benign and pathogenic variants. The red dashed diagonal indicates random classification. d) Compares the mean minimum heavy-atom distances of benign and pathogenic variants to the membrane, GTPase, and GTP. Error bars show SD. e) Shows the corresponding distance distributions, with boxes spanning the middle 50% of values, center lines marking the median, and whiskers extending over the non-outlier range. Structural distance analyses used 76 trajectory frames sampled at 1-ns intervals over 75 ns. Per-frame minimum heavy-atom distances were calculated with GROMACS 2021[12]. These distances were used directly to determine contact occupancy in panel (a) and were averaged across frames for each residue for the analyses in panels (c–e).

Table S1. Performance of missense variant predictors on the clinical SynGAP variant dataset using default thresholds with standard deviations.

Full SynGAP-α2 sequence (res. 1-1343)

Prediction method [(1)]	Recall [(2)]	Balanced accuracy [(2)]	F1 Score [(2)]	MCC [(2)]	AUPRC [(2)]	ROC-AUC [(3)]	Coverage % [(4)]
AlphaMissense	0.939 ± 0.029	0.818 ± 0.025	0.762 ± 0.027	0.615 ± 0.048	0.917 ± 0.025	***0.937 ± 0.022***	88
ESM1b	0.968 ± 0.023	***0.866 ± 0.022***	0.797 ± 0.027	0.694 ± 0.042	0.849 ± 0.042	0.928 ± 0.018	94
Provean	0.924 ± 0.032	0.839 ± 0.024	0.765 ± 0.028	0.641 ± 0.046	0.839 ± 0.039	0.917 ± 0.020	***100***
Revel	0.728 ± 0.055	0.808 ± 0.031	0.745 ± 0.041	0.625 ± 0.059	0.830 ± 0.036	0.896 ± 0.022	***100***
EVE	0.967 ± 0.023	0.830 ± 0.031	0.836 ± 0.029	0.678 ± 0.057	0.765 ± 0.059	0.882 ± 0.027	51
Foldetta	0.885 ± 0.045	0.865 ± 0.049	***0.910 ± 0.030***	***0.700 ± 0.097***	***0.926 ± 0.024***	0.876 ± 0.037	23
FoldX-MD	0.901 ± 0.042	0.799 ± 0.071	***0.910 ± 0.030***	0.579 ± 0.132	0.922 ± 0.025	0.867 ± 0.039	24
CADD	0.903 ± 0.043	0.855 ± 0.028	0.784 ± 0.035	0.674 ± 0.055	0.753 ± 0.043	0.849 ± 0.028	72
Rosetta	0.856 ± 0.050	0.838 ± 0.049	0.883 ± 0.035	0.656 ± 0.096	0.902 ± 0.028	0.846 ± 0.039	23
PolyPhen-2 Var	0.923 ± 0.033	0.703 ± 0.027	0.619 ± 0.024	0.400 ± 0.050	0.758 ± 0.040	0.825 ± 0.030	***100***
PolyPhen-2 Div	***0.985 ± 0.015***	0.650 ± 0.021	0.582 ± 0.016	0.341 ± 0.037	0.797 ± 0.036	0.792 ± 0.031	***100***
SIFT	0.881 ± 0.040	0.692 ± 0.029	0.612 ± 0.027	0.375 ± 0.055	0.808 ± 0.036	0.771 ± 0.032	***100***
PremPS	0.772 ± 0.059	0.702 ± 0.055	0.780 ± 0.045	0.401 ± 0.108	0.807 ± 0.059	0.759 ± 0.048	27
FATHMM	0.607 ± 0.061	0.672 ± 0.035	0.568 ± 0.045	0.336 ± 0.068	0.600 ± 0.050	0.701 ± 0.041	***100***

The PH-C2-GAP structural region of SynGAP1-α2 (res. 198-730)

Prediction method [(1)]	Recall [(2)]	Balanced accuracy [(2)]	F1 Score [(2)]	MCC [(2)]	AUPRC [(2)]	ROC-AUC [(3)]	Coverage % [(4)]
AlphaMissense	0.983 ± 0.017	0.754 ± 0.041	0.856 ± 0.022	0.601 ± 0.070	***0.962 ± 0.016***	***0.953 ± 0.019***	90
ESM1b	***1.000 ± 0.000***	0.716 ± 0.042	0.836 ± 0.021	0.556 ± 0.066	0.879 ± 0.040	0.876 ± 0.036	93
Provean	***1.000 ± 0.000***	0.757 ± 0.038	0.846 ± 0.020	0.613 ± 0.058	0.875 ± 0.038	0.863 ± 0.039	***100***
Revel	0.823 ± 0.052	0.750 ± 0.043	0.797 ± 0.036	0.510 ± 0.086	0.879 ± 0.032	0.844 ± 0.039	***100***
EVE	***1.000 ± 0.000***	0.729 ± 0.052	0.891 ± 0.023	0.604 ± 0.081	0.791 ± 0.061	0.833 ± 0.049	70
Foldetta	0.883 ± 0.045	***0.865 ± 0.047***	0.910 ± 0.030	**0.700 ± 0.091**	0.926 ± 0.022	0.877 ± 0.036	59
FoldX-MD	0.902 ± 0.042	0.800 ± 0.069	***0.911 ± 0.029***	0.585 ± 0.128	0.922 ± 0.023	0.867 ± 0.038	61
CADD	0.936 ± 0.037	0.768 ± 0.049	0.851 ± 0.030	0.587 ± 0.093	0.803 ± 0.042	0.751 ± 0.053	67
Rosetta	0.854 ± 0.052	0.839 ± 0.047	0.882 ± 0.035	0.657 ± 0.092	0.902 ± 0.026	0.847 ± 0.039	57
PolyPhen-2 Var	0.947 ± 0.030	0.647 ± 0.040	0.774 ± 0.024	0.379 ± 0.086	0.859 ± 0.033	0.786 ± 0.046	***100***
PolyPhen-2 Div	***1.000 ± 0.000***	0.626 ± 0.034	0.777 ± 0.016	0.398 ± 0.061	0.884 ± 0.026	0.677 ± 0.052	***100***
SIFT	0.911 ± 0.037	0.711 ± 0.044	0.800 ± 0.029	0.472 ± 0.089	0.922 ± 0.022	0.835 ± 0.042	***100***
PremPS	0.770 ± 0.061	0.700 ± 0.054	0.778 ± 0.043	0.399 ± 0.107	0.805 ± 0.054	0.758 ± 0.047	68
FATHMM	0.634 ± 0.065	0.561 ± 0.050	0.627 ± 0.047	0.123 ± 0.100	0.706 ± 0.048	0.636 ± 0.058	***100***

Values are reported as bootstrapped means ± standard deviation (SD). Confidence intervals for the bootstrapped balanced accuracy estimates and DeLong significance tests for ROC-AUC comparisons are presented in Figures 1 and S2. The same metrics are given with confidence intervals in Table 1. The best-performing method for each metric, based only on the bootstrapped mean value, is shown in bold italics.

(1) Prediction methods: Methods evaluated include AlphaMissense[35], ESM1b[34], PolyPhen-2 (HumDiv and HumVar)[33], FATHMM[36] SIFT[30], PROVEAN[29], REVEL[39], CADD[37], EVE[38], FoldX-MD[43], Rosetta[15,21], Foldetta[27], and PremPS[28].

Pathogenic and likely pathogenic ClinVar variants were combined and treated as positive cases, whereas benign and likely benign variants were combined and treated as negative cases.

(2) Threshold-dependent performance metrics: Recall (sensitivity), balanced accuracy, F1 score, Matthews correlation coefficient (MCC), and area under the precision-recall curve (AUPRC) were calculated using the default classification thresholds provided by each method. Definitions of all metrics are provided in Eqs. 1-7 of the SI. The default thresholds are given in Table S2.

(3) ROC-AUC: The area under the receiver operating characteristic curve (ROC-AUC) evaluates the ability of a method to discriminate between pathogenic and benign variants across all possible classification thresholds and is therefore independent of the default threshold used for classification.

(4) Coverage %: Coverage or prediction rate denotes the proportion of SynGAP missense variants for which a method generated predictions. Rates are reported both for the entire SynGAP protein (residues 1-1343) and for the N-terminal structural region (residues 198-730). For folding stability methods (FoldX-MD, Rosetta, Foldetta, and PremPS), predictions were restricted to the N-terminal SynGAP structural model (residues 198-730; Figure 1b). Consequently, their performance metrics are directly comparable only within this subset, which comprises ~30% of the SynGAP missense variants in the SGM database.

Table S2. In silico pathogenicity predictor thresholds applied in this study

Method	Pathogenic direction(1)	Default / Recommended(2)	Optimized Single(3)	Optimized Dual(4)	ACMG Moderate(5)	ACMG Strong(6)
AlphaMissense	Higher	0.340 / 0.564	0.7866	0.7832 / 0.9776	0.099 / 0.906	0.099 / 0.990
ESM1b	Lower	-7.0/ -8.0	-7.527	-7.474 / -11.218	-3.1 / -12.2	-3.1 / -24.0
REVEL	Higher	0.500	0.466	0.204 / 0.482	0.183 / 0.773	0.016 / 0.932
FoldX	Higher	-2.0/ -0.5<br>0.5/ 2.0	1.870	N/A	N/A	N/A
Rosetta	Higher	-2.0/ -0.5<br>0.5 / 2.0	1.520	N/A	N/A	N/A
Foldetta	Higher	-2.0/ -0.5<br>0.5 / 2.0	2.110	N/A	N/A	N/A
PremPS	Higher	-1.0/ -0.5<br>0.5 / 1.0	1.590	N/A	N/A	N/A
PROVEAN	Lower	-2.5	-4.240	-2.44 / -4.53	N/A	N/A
FATHMM	Lower	2.5	1.640	2.61 / 1.64	4.69 / -5.04	N/A
PolyPhen-2 HumDiv	Higher	0.452	0.996	0.935 / 0.997	N/A	N/A
PolyPhen-2 HumVar	Higher	0.447	0.750	0.863 / 0.992	0.009 / 0.999	N/A
SIFT	Lower	0.05	0.060	0.070 / 0.01	0.327 / 0.000	N/A
EVE	Higher	0.35 / 0.65	0.644643	0.641151 /<br>0.812466	N/A	N/A
CADD	Higher	26.1 / 29.3	27.600	26.1 / 29.3	17.3 / 25.3	0.15 / 28.1

Yonden J based optimization was done with minimum 80 % coverage for test set variants. Note that the optimized thresholds are specific to the SynGAP clinical dataset and should be reassessed as additional classified variants become available. Currently, only the dual optimized thresholds for AphaMissense and CADD are implemented in the SGM server.

(1) Pathogenic direction: Higher = Scores above the pathogenic threshold are pathogenic. Lower = Scores below the pathogenic threshold are pathogenic.

(2) Default / Recommended thresholds were applied or recommended in the original publication presenting the method. CADD requires gene-specific calibration, so its Default and Optimized Dual thresholds are the same.

(3) Optimized Single: Single thresholds divide scores directly into benign and pathogenic classes, without an uncertain interval.

(4) Optimized Dual: Dual thresholds define separate benign and pathogenic boundaries, with intermediate scores remaining uncertain or uncalled.

(5) ACMG Moderate: Modified from Bergquist et al.[47] and Pejaver et al.[48]

(5) ACMG Strong: Modified from Bergquist et al.[47] and Pejaver et al.[48]

Table S3. Comparison of differently calibrated thresholds for the in silico predictor methods regarding MCC and prediction coverage

MCC [(1)]

Method	Default	ACMG Mod	ACMG Strong	Optimized single	Optimized dual
AlphaMissense	0.613	0.704	***0.925***	0.772	0.905
CADD	**0.672**	0.244	N/A	0.558	**0.672**
ESM1b	0.691	0.801	N/A	0.680	**0.842**
EVE	0.671	N/A	N/A	0.660	**0.766**
FATHMM	0.333	N/A	N/A	0.475	**0.495**
Foldetta	0.694	N/A	N/A	0.713	N/A
FoldX-MD	0.577	N/A	N/A	0.742	N/A
PolyPhen-2 Div	0.342	N/A	N/A	0.485	**0.531**
PolyPhen-2 Var	0.398	**0.830**	N/A	0.459	0.571
PremPS	0.397	N/A	N/A	0.511	N/A
Provean	0.638	N/A	N/A	0.703	**0.824**
Revel	0.621	**0.843**	N/A	0.665	0.716
Rosetta	0.651	N/A	N/A	0.626	N/A
SIFT	0.372	**0.558**	N/A	0.407	0.473

Coverage % [(2)]

Method	Default	ACMG Mod	ACMG Strong	Optimized single	Optimized dual
AlphaMissense	88	41	24	**100**	79
CADD	72	57	29	**100**	72
ESM1b	94	27	12	**100**	79
EVE	51	N/A	N/A	78	61
FATHMM	**100**	9	N/A	**100**	80
Foldetta	23	N/A	N/A	40	N/A
FoldX-MD	24	N/A	N/A	40	N/A
PolyPhen-2 Div	**100**	N/A	N/A	**100**	77
PolyPhen-2 Var	**100**	17	N/A	**100**	80
PremPS	26	N/A	N/A	40	N/A
Provean	**100**	N/A	N/A	**100**	76
Revel	**100**	52	1	**100**	70
Rosetta	23	N/A	N/A	40	N/A
SIFT	**100**	43	N/A	**100**	80

[(1)] MCC is described in detail in "*Equations for assessing prediction accuracy and relatedness*" section of the SI. For each prediction method, the highest MCC and coverage (%) achieved across the evaluated thresholding strategies are shown in bold. The overall highest MCC is indicated by underlined italics (AlphaMissense + ACMG Strong).

[(2)] Coverage (%) represents the proportion of missense variants receiving a callable classification (Benign or Pathogenic) relative to variants classified as Uncertain or simply missing prediction. Coverage was calculated against all missense variants in the SGM database (N = 7,892). For each method, the highest coverage value obtained across the evaluated thresholding strategies is shown in bold.

Table S4. Correlation between different pathogenicity prediction methods.

Compared prediction methods	ϕ coefficient [1]	p-value	R^2 [2]	p-value
AlphaMissense & ESM1b	0.67	4.27×10^{-67}	0.48	2.19×10^{-117}
AlphaMissense & EVE	0.77	5.56×10^{-49}	0.53	6.25×10^{-101}
AlphaMissense & REVEL	0.48	8.00×10^{-37}	0.39	1.16×10^{-87}
AlphaMissense & PolyPhen-2 HumDiv	0.43	2.18×10^{-29}	0.23	1.11×10^{-48}
AlphaMissense & PolyPhen-2 HumVar	0.45	4.74×10^{-33}	0.28	1.21×10^{-60}
AlphaMissense & FATHMM	0.2	6.06×10^{-8}	0.06	1.19×10^{-11}
AlphaMissense & SIFT	0.31	6.80×10^{-16}	0.09	7.03×10^{-19}
AlphaMissense & PROVEAN	0.64	9.21×10^{-64}	0.49	5.87×10^{-119}
ESM1b & EVE	***0.8***	1.69×10^{-56}	0.6	2.03×10^{-122}
ESM1b & REVEL	0.61	1.03×10^{-63}	0.56	1.18×10^{-145}
ESM1b & PolyPhen-2 HumDiv	0.4	5.86×10^{-28}	0.21	1.60×10^{-42}
ESM1b & PolyPhen-2 HumVar	0.41	5.51×10^{-29}	0.26	6.51×10^{-54}
ESM1b & FATHMM	0.3	9.16×10^{-17}	0.13	1.43×10^{-25}
ESM1b & SIFT	0.24	1.96×10^{-11}	0.11	1.63×10^{-21}
ESM1b & PROVEAN	0.68	8.92×10^{-79}	0.57	4.22×10^{-148}
EVE & REVEL	0.57	7.13×10^{-31}	0.45	7.46×10^{-80}
EVE & PolyPhen-2 HumDiv	0.59	4.75×10^{-32}	0.31	1.14×10^{-49}
EVE & PolyPhen-2 HumVar	0.59	2.94×10^{-32}	0.35	7.61×10^{-59}
EVE & FATHMM	0.34	1.39×10^{-11}	0.05	1.69×10^{-8}
EVE & SIFT	0.63	1.64×10^{-36}	0.28	5.02×10^{-45}
EVE & PROVEAN	0.89	5.52×10^{-71}	0.57	2.29×10^{-113}
REVEL & PolyPhen-2 HumDiv	0.28	3.34×10^{-15}	0.18	4.52×10^{-37}
REVEL & PolyPhen-2 HumVar	0.31	5.33×10^{-19}	0.25	1.38×10^{-52}
REVEL & FATHMM	0.39	4.85×10^{-29}	0.21	1.46×10^{-42}
REVEL & SIFT	0.21	2.76×10^{-9}	0.06	7.29×10^{-12}
REVEL & PROVEAN	0.53	4.76×10^{-52}	0.49	5.35×10^{-120}
PolyPhen-2 HumDiv & PolyPhen-2 HumVar	0.74	5.96×10^{-98}	***0.78***	2.43×10^{-268}
PolyPhen-2 HumDiv & FATHMM	0.2	8.31×10^{-9}	0.06	3.03×10^{-12}
PolyPhen-2 HumDiv & SIFT	0.21	1.08×10^{-9}	0.08	4.62×10^{-16}
PolyPhen-2 HumDiv & PROVEAN	0.42	2.36×10^{-33}	0.23	3.56×10^{-47}
PolyPhen-2 HumVar & FATHMM	0.29	1.63×10^{-16}	0.12	4.56×10^{-24}
PolyPhen-2 HumVar & SIFT	0.23	7.45×10^{-11}	0.06	2.39×10^{-13}
PolyPhen-2 HumVar & PROVEAN	0.45	3.00×10^{-37}	0.29	5.19×10^{-61}
FATHMM & SIFT	<u>0.08</u>	0.018	<u>0</u>	0.116
FATHMM & PROVEAN	0.38	4.43×10^{-27}	0.12	7.85×10^{-25}
SIFT & PROVEAN	0.32	8.78×10^{-20}	0.15	7.41×10^{-30}

The highest value is highlighted in bold and italics, in contrast, the lowest value is underlined.

[1] Phi (ϕ) coefficient measures the strength and direction of the association between two binary variables represented by the two prediction methods being compared. See Eq. 8 below in the SI. The corresponding p-value was calculated using a Pearson chi-square test of independence on the same 2 × 2 contingency table and tests the null hypothesis that the two classifications are independent. $P < 0.05$ was considered statistically significant.

[2] R-squared (R^2) measures the strength of the linear relationship between pairs of predicted values. See Eq. 9 below in the SI. The corresponding p-value was obtained from the Pearson correlation significance test and tests the null hypothesis that the underlying linear correlation is zero. $P < 0.05$ was considered statistically significant.

Table S5. Correlation between different pathogenicity prediction methods against the folding stability methods.

Compared prediction methods	ϕ coefficient [(1)]	p-value	R^2 [(2)]	p-value
FoldX-MD & AlphaMissense	0.50	1.89×10^{-11}	0.18	2.16×10^{-16}
FoldX-MD & ESM1b	0.43	3.80×10^{-9}	0.23	2.70×10^{-20}
FoldX-MD & EVE	0.56	2.12×10^{-11}	0.23	2.20×10^{-19}
FoldX-MD & REVEL	0.48	8.40×10^{-12}	0.20	1.33×10^{-17}
FoldX-MD & PolyPhen-2 HumDiv	0.26	2.40×10^{-4}	0.04	1.60×10^{-4}
FoldX-MD & PolyPhen-2 HumVar	0.34	1.06×10^{-6}	0.06	2.42×10^{-6}
FoldX-MD & FATHMM	0.14	0.042	0.01	0.120
FoldX-MD & SIFT	0.44	5.36×10^{-10}	0.09	2.20×10^{-8}
FoldX-MD & PROVEAN	0.39	3.69×10^{-8}	0.18	8.01×10^{-16}
FoldX-MD & Rosetta	0.89	1.31×10^{-26}	0.59	3.39×10^{-66}
FoldX-MD & Foldetta	***1***	5.99×10^{-40}	0.87	1.85×10^{-147}
FoldX-MD & PremPS	0.63	3.20×10^{-14}	0.24	1.97×10^{-21}
Rosetta & AlphaMissense	0.48	1.66×10^{-10}	0.20	8.54×10^{-18}
Rosetta & ESM1b	0.40	5.02×10^{-8}	0.22	3.22×10^{-20}
Rosetta & EVE	0.50	1.07×10^{-9}	0.19	1.49×10^{-16}
Rosetta & REVEL	0.49	6.27×10^{-12}	0.20	5.01×10^{-18}
Rosetta & PolyPhen-2 HumDiv	0.21	0.004	0.05	7.10×10^{-5}
Rosetta & PolyPhen-2 HumVar	0.29	5.26×10^{-5}	0.06	6.45×10^{-6}
Rosetta & FATHMM	0.06	0.394	0.01	0.128
Rosetta & SIFT	0.39	7.54×10^{-8}	0.07	1.59×10^{-6}
Rosetta & PROVEAN	0.40	2.62×10^{-8}	0.15	2.73×10^{-13}
Rosetta & Foldetta	0.99	1.62×10^{-37}	***0.90***	4.25×10^{-169}
Rosetta & PremPS	0.66	3.57×10^{-15}	0.30	3.30×10^{-28}
Foldetta & AlphaMissense	0.51	3.65×10^{-12}	0.22	1.87×10^{-19}
Foldetta & ESM1b	0.43	1.57×10^{-9}	0.25	4.17×10^{-23}
Foldetta & EVE	0.54	1.71×10^{-11}	0.24	4.03×10^{-20}
Foldetta & REVEL	0.47	9.41×10^{-12}	0.23	2.87×10^{-20}
Foldetta & PolyPhen-2 HumDiv	0.20	0.004	0.05	3.51×10^{-5}
Foldetta & PolyPhen-2 HumVar	0.26	1.51×10^{-4}	0.07	8.95×10^{-7}
Foldetta & FATHMM	0.07	0.308	0.01	0.102
Foldetta & SIFT	0.36	1.19×10^{-7}	0.09	3.58×10^{-8}
Foldetta & PROVEAN	0.41	3.06×10^{-9}	0.18	2.63×10^{-16}
Foldetta & PremPS	0.67	4.04×10^{-17}	0.31	1.39×10^{-28}
PremPS & AlphaMissense	0.46	5.50×10^{-11}	0.27	6.02×10^{-25}
PremPS & ESM1b	0.51	6.68×10^{-14}	0.31	2.93×10^{-29}
PremPS & EVE	0.55	1.97×10^{-12}	0.25	4.65×10^{-21}
PremPS & REVEL	0.38	6.30×10^{-9}	0.21	4.17×10^{-19}
PremPS & PolyPhen-2 HumDiv	0.29	9.87×10^{-6}	0.12	4.50×10^{-11}
PremPS & PolyPhen-2 HumVar	0.27	4.14×10^{-5}	0.12	3.50×10^{-11}
PremPS & FATHMM	0.05	0.458	0.02	0.007
PremPS & SIFT	0.29	1.61×10^{-5}	0.14	1.81×10^{-12}
PremPS & PROVEAN	0.51	1.31×10^{-14}	0.22	1.57×10^{-19}

The highest value is highlighted in bold and italics, in contrast, the lowest value is underlined.

[(1)] Phi (ϕ) coefficient measures the strength and direction of the association between two binary variables represented by the two prediction methods being compared. See Eq. 8 below in the SI. The corresponding p-value was calculated using a Pearson chi-square test of independence on the same 2 × 2 contingency table and tests the null hypothesis that the two classifications are independent. $P < 0.05$ was considered statistically significant.

[(2)] R-squared (R^2) measures the strength of the linear relationship between pairs of predicted values. See Eq. 9 below in the SI. The corresponding p-value was obtained from the Pearson correlation significance test and tests the null hypothesis that the underlying linear correlation is zero. $P < 0.05$ was considered statistically significant.

Table S6. Density per residue and relative density of AlphaMissense categorized SynGAP missense variants by domain.

Location (1)	All	Pathogenic (2)		Benign (2)		Unclear (2)	
	Density per residue (3)	Density per residue (3)	Relative density (4)	Density per residue (3)	Relative density (4)	Density per residue (3)	Relative density (4)
PH	5.89	4.57	0.78	0.73	0.12	0.59	0.10
C2	5.73	3.39	0.59	1.80	0.31	0.55	0.10
GAP	5.92	4.42	0.75	0.90	0.15	0.60	0.10
NPxF motif	6.11	5.67	0.93	0.22	0.04	0.22	0.04
SH3-binding motif	5.90	1.87	0.32	2.61	0.44	1.42	0.24
CC	5.88	4.09	0.70	1.15	0.20	0.64	0.11
Domains	5.91	4.00	0.68	1.24	0.21	0.67	0.11
Non-domain	5.88	1.96	0.33	3.09	0.53	0.83	0.14
Ordered	0.48	0.32	0.68	0.10	0.21	0.05	0.11
Disordered	0.52	0.18	0.35	0.27	0.51	0.07	0.14
All	5.88	2.99	0.51	2.16	0.37	0.73	0.12

(1) Location as residue ranges: PH (199-254), C2 (256-409), GAP (411-724), and CC (1170-1270) domains as well as SH3-binding motif (785-815) or NPxF motif (924-932). Non-domain refers to all other residues in the protein.

(2) AlphaMissense default scoring used in the categorization into Pathogenic, Benign and Unclear categories.

(3) Density per residue: The variant density in relation to the total residue count of the location.

(4) Relative density: The variant density in relation to the total variant count of the location.

Table S7. AlphaMissense score strength per SynGAP domain.

Variant set (1)	Location (2)	Pathogenic (3)	Benign (3)	Unclear (3)	All
All established and likely theoretical variants	PH	0.93	0.17	0.45	0.79
	C2	0.89	0.15	0.45	0.62
	GAP	0.90	0.17	0.46	0.75
	SH3-binding motif	0.76	0.14	0.45	0.41
	NPxF motif	0.93	0.26	0.50	0.89
	CC	0.89	0.18	0.46	0.71
	Non-domain	0.84	0.15	0.45	0.42
	All	0.88	0.16	0.45	0.56
ClinVar only	All	0.87	0.15	0.44	0.51
gnomAD only	All	0.83	0.15	0.45	0.41
Theoretical only	All	0.88	0.16	0.45	0.59

(1) The AlphaMissense scores were calculated for all missense variants included in the SGM server, including all verified variants from ClinVar and gnomAD, as well as likely theoretical variants emerging from single-base substitutions. Alternatively, the scores were averaged only for ClinVar, gnomAD or theoretical variants.

(2) Location as residue ranges: PH (199-254), C2 (256-409), GAP (411-724), and CC (1170-1270) domains as well as SH3-binding motif (785-815) or NPxF motif (924-932). Non-domain refers to all other residues in the protein.

(3) AlphaMissense default scoring used in the categorization into Pathogenic, Benign and Unclear categories.

Table S8. AlphaMissense score strength per SynGAP gnomAD allele count.

Allele Count Bin[(1)]	Variant Count[(2)]	AlphaMissense Score				
		AVE	MED	MAX	MIN	STDEV
1–10	891	0.43	0.32	1.00	0.04	0.33
11–100	88	0.31	0.19	0.95	0.05	0.26
>100	7	0.14	0.08	0.50	0.07	0.16
All	986	0.42	0.30	1.00	0.04	0.32

[(1)] The gnomAD missense variants (retrieved on 20/05/2024) were divided into three bins based on allele count: 1-9, 10-99, and ≥100.

[(2)] Variant count per allele count bin.

Table S9. PSMutPred prediction scores for phase separation impacting SynGAP missense variants.

Location [(1)]	PSMutPred Average scores			No. of PS Impact Variants [(5)]
	IP RF [(2)]	IP SVR [(3)]	SP RF [(4)]	
PH	0.52	0.39	0.43	2
C2	0.53	0.37	0.34	14
GAP	0.52	0.46	0.27	5
SH3-binding motif	0.53	0.46	0.18	4
CC	0.52	0.37	0.14	4
Non-domain	0.53	0.36	0.31	41
All	0.53	0.38	0.30	70

[(1)] Location as residue ranges: PH (199-254), C2 (256-409), GAP (411-724), and CC (1170-1270) domains as well as SH3-binding motif (785-815) or NPxF motif (924-932). Non-domain refers to all other residues in the protein.

[(2)] PSMutPred score IP RF (Impact Prediction – Random Forest): Used in binary classification of missense variants as phase separation (PS) impacting (> 0.5) or not impacting (< 0.5).

[(3)] PSMutPred score IP SVR (Impact Prediction – Support Vector Regression): Estimates the strength of PS change caused by a missense mutation.

[(4)] PSMutPred score SP RF (Strength Prediction – Random Forest): Variants with a score >0.5 predicted to strengthen PS; variants with a score below 0.5 predicted to weaken PS.

[(5)] Number of PS-impacted missense variants based on PSMutPred score IP RF (> 0.5): Count all variants where IP RF score is greater than 0.5. Those variants not meeting this criterion were not used in the average calculations.

Table S10. Proximity-based phase separation impact mutation clustering of SynGAP.

Cluster # (1)	Cluster residue range (2)	PSMutPred Average scores			Cluster size (6)
		IP RF AVE (3)	IP SVR AVE (4)	SP RF AVE (5)	
1	3-73	***0.55***	0.33	***0.49***	8
2	100-185	***0.53***	0.33	0.25	16
3	235-239	***0.52***	0.39	0.43	2
4	282-331	***0.52***	0.32	0.30	6
5	362-397	***0.53***	0.37	0.36	8
6	460	***0.51***	***0.52***	0.12	1
7	524-534	***0.52***	0.36	0.25	2
8	587-604	***0.50***	0.12	***0.46***	2
9	752-821	***0.52***	0.29	0.34	12
10	872-892	***0.52***	0.19	***0.47***	2
11	984-994	***0.51***	***0.49***	0.16	2
12	1043	***0.51***	***0.56***	0.41	1
13	1083	***0.51***	0.43	0.19	1
14	1130-1135	***0.51***	***0.51***	0.19	2
15	1165-1183	***0.53***	0.29	0.23	3
16	1219-1237	***0.51***	0.41	0.11	2

(1) Proximity-based clustering done using ≤25 aa bin. PSMutPred IP RF score (>0.5) used to select missense variants affecting phase separation (PS).

(2) Phase separation impact mutation cluster residue range.

(3) PSMutPred score IP RF (Impact Prediction – Random Forest): Estimates the strength of PS change caused by a missense mutation.

(4) PSMutPred score SP RF (Strengthen/Weaken Prediction – Random Forest): Predicts whether a missense mutation strengthens or weakens phase separation propensity. Variants are not selected in clusters based on this score.

(5) PSMutPred score IP SVR (Impact Prediction – Support Vector Regression): Estimates the strength of PS change caused by a missense mutation. Variants are not selected in clusters based on this score.

(6) The number of impact mutation sites within each cluster. Note that one site can have multiple alternative PS impacting mutations but those were not calculated here.

Table S11. Phosphosites of SynGAP-α2 isoform.

**Residue** [(1)]	**LTP** [(2)]	**HTP** [(3)]	**Total**	**Residue** [(1)]	**LTP** [(2)]	**HTP** [(3)]	**Total**	**Residue** [(1)]	**LTP** [(2)]	**HTP** [(3)]	**Total**
S7*	0	1	1	Y758	0	45	45	***S985***	0	2	2
S12	0	4	4	***S765***	0	3	3	***S1003***	0	5	5
S17*	0	4	4	S766*	0	8	8	S1005	0	3	3
Y18	0	69	69	***S780***	1	6	7	Y1006	0	7	7
Y34	0	63	63	***S788****	2	23	25	S1053	0	1	1
S37*	0	14	14	***T790***	0	8	8	***S1057***	0	1	1
Y39	0	88	88	***Y807***	0	32	32	S1073	1	6	7
T85	0	1	1	S809	0	4	4	***S1079***	0	3	3
S87-p	0	13	13	S817*	1	3	4	***S1087***	0	7	7
Y100*	0	25	25	***Y820****	0	61	61	***S1088***	0	4	4
S105*	0	2	2	T822	0	3	3	S1094	0	8	8
S109	0	3	3	S823	0	4	4	***S1098***	0	6	6
Y116*	0	18	18	S824	0	2	2	Y1099	0	18	18
S117	0	15	15	***S825***	0	11	11	***S1108***	1	3	4
T153	0	1	1	T828	0	6	6	***S1110***	0	1	1
S155	0	2	2	***S836***	1	2	3	S1114-p	1	8	9
S176	0	1	1	***S840-p***	1	2	3	***S1118***	0	3	3
Y291-p	0	1	1	***S842-p***	1	3	4	***S1121***	0	5	5
S302	0	1	1	S857	0	1	1	***S1132-p***	0	1	1
Y342	0	33	33	***S861***	0	1	1	***S1138***	2	2	4
S371	0	3	3	S872*	0	1	1	S1151-p	0	1	1
S374	0	3	3	***S873***	0	3	3	S1165*	0	8	8
S379*	1	3	4	***S876***	0	1	1	Y1183-p	0	3	3
S385	1	1	2	S892*	0	5	5	S1184-p	0	2	2
S449-p	1	2	3	***S895***	1	7	8	S1186-p	0	2	2
T458	0	1	1	S898	0	3	3	S1190-p	0	2	2
S466	1	2	3	***T900***	0	1	1	Y1198	0	6	6
Y518	0	1	1	***S907-p***	0	2	2	S1204	0	11	11
S571	0	2	2	T912-p	0	4	4	Y1219	0	6	6
T723	0	1	1	T913-p	0	2	2	S1225	0	6	6
S735	0	1	1	***Y982***	0	36	36	Y1237	0	6	6
S752*	0	6	6	***S983***	0	1	1	S1261-p	0	2	2

(1) Phosphosites for S, T and Y acquired from the PhophositePlus (www.phosphosite.org; 09/30/2025). Duplicates and other PTMs removed for clarity.

(2) LTP (Low-Throughput Papers): Non-mass spectroscopy methods.

(3) HTP (High-Throughput Papers): Mass spectroscopy methods.

* Impact mutations based on PSMutPred IP RF prediction.

Those residues with matching missense variants in ClinVar were highlighted in bold and italics.

Those phosphosites present directly in human-derived data (instead of rat or mouse) were assigned small letter p (e.g., Y291-p).

Table S12. Proximity-based phosphosite clustering of SynGAP.

Cluster # (1)	Cluster residue range (2)	Evidence sum (3)	Cluster size (4)
1	7-39	243	7
2	85-117	77	7
3	153-176	4	3
4	291-302	2	2
5	342	33	1
6	371-385	12	4
7	449-466	7	3
8	518	1	1
9	571	2	1
10	723-913	273	33
11	982-1006	54	6
12	1053-1261	139	29

(1) Proximity-based clustering done using ≤25 aa bin. See Table S13 for the original non-clustered PhosphoSitePlus data.

(2) Phosphosite cluster residue range.

(3) Phosphosite evidence, acquired via PhosphoSitePlus combines LTP and HTP for each residue.

(4) The number of phosphosites within each cluster.

References

1. Ali, A. E., Li, L.-L., Courtney, M. J., Pentikäinen, O. T. & Postila, P. A. Atomistic simulations reveal impacts of missense mutations on the structure and function of SynGAP1. *Brief. Bioinform.* **25**, (2024).

2. Jumper, J. *et al.* Highly accurate protein structure prediction with AlphaFold. *Nature* **596**, 583–589 (2021).

3. Šali, A. & Blundell, T. L. Comparative Protein Modelling by Satisfaction of Spatial Restraints. *J. Mol. Biol.* **234**, 779–815 (1993).

4. Ali, A. E., Li, L.-L., Courtney, M. J., Pentikäinen, O. T. & Postila, P. A. Atomistic Simulations Reveal Impacts of Missense Mutations on the Structure and Function of SynGAP1. *Briefings in Bioinformatics* https://doi.org/10.1093/bib/bbae458 (2024) doi:10.1093/bib/bbae458.

5. Case, D. A. *et al.* Amber 2023, University of California, San Francisco.

6. Case, D. A. *et al.* AmberTools. *J. Chem. Inf. Model.* **63**, 6183–6191 (2023).

7. Chen, E. A. & Porter, L. L. SSDraw: software for generating comparative protein secondary structure diagrams. https://doi.org/10.1101/2023.08.25.554905 doi:10.1101/2023.08.25.554905.

8. Lehtonen, J. V *et al. BODIL: A Molecular Modeling Environment for Structure-Function Analysis and Drug Design*. http://www.abo.fi/fak/mnf/bkf/research/johnson/bodil.html.

9. Lomize, M. A., Lomize, A. L., Pogozheva, I. D. & Mosberg, H. I. OPM: orientations of proteins in membranes database. *Bioinformatics* **22**, 623–625 (2006).

10. Yung, Y. L. *et al.* Site-directed mutagenesis shows the significance of interactions with phospholipids and the G-protein OsYchF1 for the physiological functions of the rice GTPase-activating protein 1 (OsGAP1). *Journal of Biological Chemistry* **290**, 23984–23996 (2015).

11. Jo, S., Kim, T., Iyer, V. G. & Im, W. CHARMM-GUI: a web-based graphical user interface for CHARMM. *Journal of computational chemistry* vol. 29 1859–1865 Preprint at https://doi.org/10.1002/jcc.20945 (2008).

12. Lindahl, Abraham, Hess & der Spoel, van. GROMACS 2021 Source code. Preprint at https://doi.org/10.5281/zenodo.4457626 (2021).

13. Scheffzek, K. *et al.* The Ras-RasGAP Complex: Structural Basis for GTPase Activation and Its Loss in Oncogenic Ras Mutants. *Science (1979).* **277**, 333–339 (1997).

14. Schymkowitz, J. *et al.* The FoldX web server: An online force field. *Nucleic Acids Res.* **33**, (2005).

15. Sora, V. *et al.* RosettaDDGPrediction for high-throughput mutational scans: From stability to binding. *Protein Science* **32**, (2023).

16. Barnes, J. E., Lund-Andersen, P. K., Patel, J. S. & Ytreberg, F. M. The effect of mutations on binding interactions between the SARS-CoV-2 receptor binding domain and neutralizing antibodies B38 and CB6. *Sci. Rep.* **12**, (2022).

17. Frenz, B. *et al.* Prediction of Protein Mutational Free Energy: Benchmark and Sampling Improvements Increase Classification Accuracy. *Front. Bioeng. Biotechnol.* **8**, (2020).

18. Leaver-Fay, A. *et al.* Rosetta3: An object-oriented software suite for the simulation and design of macromolecules. in *Methods in Enzymology* vol. 487 545–574 (Academic Press Inc., 2011).

19. Fleishman, S. J. *et al.* Rosettascripts: A scripting language interface to the Rosetta Macromolecular modeling suite. *PLoS One* **6**, (2011).

20. Chaudhury, S., Lyskov, S. & Gray, J. J. PyRosetta: A script-based interface for implementing molecular modeling algorithms using Rosetta. *Bioinformatics* vol. 26 689–691 Preprint at https://doi.org/10.1093/bioinformatics/btq007 (2010).

21. Leman, J. K. *et al.* Macromolecular modeling and design in Rosetta: recent methods and frameworks. *Nature Methods* vol. 17 665–680 Preprint at https://doi.org/10.1038/s41592-020-0848-2 (2020).

22. Tyka, M. D. *et al.* Alternate states of proteins revealed by detailed energy landscape mapping. *J. Mol. Biol.* **405**, 607–618 (2011).

23. Valanciute, A. *et al.* Accurate protein stability predictions from homology models. *Comput. Struct. Biotechnol. J.* **21**, 66–73 (2023).

24. Alford, R. F. *et al.* The Rosetta All-Atom Energy Function for Macromolecular Modeling and Design. *J. Chem. Theory Comput.* **13**, 3031–3048 (2017).

25. Shapovalov, M. V. & Dunbrack, R. L. A smoothed backbone-dependent rotamer library for proteins derived from adaptive kernel density estimates and regressions. *Structure* **19**, 844–858 (2011).

26. Park, H. *et al.* Simultaneous Optimization of Biomolecular Energy Functions on Features from Small Molecules and Macromolecules. *J. Chem. Theory Comput.* **12**, 6201–6212 (2016).

27. Gerasimavicius, L., Livesey, B. J. & Marsh, J. A. Correspondence between functional scores from deep mutational scans and predicted effects on protein stability. *Protein Science* **32**, (2023).

28. Chen, Y. *et al.* PremPS: Predicting the impact of missense mutations on protein stability. *PLoS Comput. Biol.* **16**, (2020).

29. Choi, Y., Sims, G. E., Murphy, S., Miller, J. R. & Chan, A. P. Predicting the Functional Effect of Amino Acid Substitutions and Indels. *PLoS One* **7**, (2012).

30. Sim, N. L. *et al.* SIFT web server: Predicting effects of amino acid substitutions on proteins. *Nucleic Acids Res.* **40**, (2012).

31. Landrum, M. J. *et al.* ClinVar: improving access to variant interpretations and supporting evidence. *Nucleic Acids Res.* **46**, D1062–D1067 (2018).

32. Sayers, E. W. *et al.* Database resources of the national center for biotechnology information. *Nucleic Acids Res.* **50**, D20–D26 (2022).

33. Adzhubei, I., Jordan, D. M. & Sunyaev, S. R. Predicting functional effect of human missense mutations using PolyPhen-2. *Curr. Protoc. Hum. Genet.* https://doi.org/10.1002/0471142905.hg0720s76 (2013) doi:10.1002/0471142905.hg0720s76.

34. Brandes, N., Goldman, G., Wang, C. H., Ye, C. J. & Ntranos, V. Genome-wide prediction of disease variant effects with a deep protein language model. *Nat. Genet.* **55**, 1512–1522 (2023).

35. Cheng, J. *et al.* Accurate proteome-wide missense variant effect prediction with AlphaMissense. *Science (1979).* **381**, (2023).

36. Shihab, H. A. *et al.* Predicting the Functional, Molecular, and Phenotypic Consequences of Amino Acid Substitutions using Hidden Markov Models. *Hum. Mutat.* **34**, 57–65 (2013).

37. Rentzsch, P., Witten, D., Cooper, G. M., Shendure, J. & Kircher, M. CADD: Predicting the deleteriousness of variants throughout the human genome. *Nucleic Acids Res.* **47**, D886–D894 (2019).

38. Frazer, J. *et al.* Disease variant prediction with deep generative models of evolutionary data. *Nature* **599**, 91–95 (2021).

39. Ioannidis, N. M. *et al.* REVEL: An Ensemble Method for Predicting the Pathogenicity of Rare Missense Variants. *Am. J. Hum. Genet.* **99**, 877–885 (2016).

40. Dosztányi, Z., Csizmok, V., Tompa, P. & Simon, I. IUPred: Web server for the prediction of intrinsically unstructured regions of proteins based on estimated energy content. *Bioinformatics* **21**, 3433–3434 (2005).

41. Piovesan, D. *et al.* MOBIDB in 2025: integrating ensemble properties and function annotations for intrinsically disordered proteins. *Nucleic Acids Res.* **53**, D495–D503 (2025).

42. Dosztányi, Z., Mészáros, B. & Simon, I. ANCHOR: Web server for predicting protein binding regions in disordered proteins. *Bioinformatics* **25**, 2745–2746 (2009).

43. Miller, C. R. *et al.* Initiating a watch list for Ebola virus antibody escape mutations. *PeerJ* **2016**, (2016).

44. Sapozhnikov, Y., Patel, J. S., Ytreberg, F. M. & Miller, C. R. Statistical modeling to quantify the uncertainty of FoldX-predicted protein folding and binding stability. *BMC Bioinformatics* **24**, (2023).

45. Hulsen, T. *DeepVenn-a Web Application for the Creation of Area-Proportional Venn Diagrams Using the Deep Learning Framework Tensorflow.Js*. https://www.deepvenn.com. doi:https://doi.org/10.48550/arXiv.2210.04597.

46. Fuchs, J. E. *et al.* Independent metrics for protein backbone and side-chain flexibility: Time scales and effects of ligand binding. *J. Chem. Theory Comput.* **11**, 851–860 (2015).

47. Bergquist, T. *et al.* Calibration of additional computational tools expands ClinGen recommendation options for variant classification with PP3/BP4 criteria. *Genetics in Medicine* **27**, (2025).

48. Pejaver, V. *et al.* Calibration of computational tools for missense variant pathogenicity classification and ClinGen recommendations for PP3/BP4 criteria. *Am. J. Hum. Genet.* **109**, 2163–2177 (2022).
